# Photo‐Modulated Proton Transport in Merocyanine Metastable‐State Photoacid Based Polymers

**DOI:** 10.1002/smll.202514786

**Published:** 2026-02-25

**Authors:** Gianni Pacella, Mira Kim, Rachael Hannah, Ryan C. Chiechi, Shirin Faraji, Giuseppe Portale

**Affiliations:** ^1^ Zernike Institute for Advanced Materials University of Groningen Groningen The Netherlands; ^2^ Institute of Theoretical and Computational Chemistry Faculty of Mathematics and Natural Sciences, Heinrich Heine University Düsseldorf Düsseldorf Germany; ^3^ Department of Chemistry & Organic and Carbon Electronics Cluster North Carolina State University Raleigh United States

**Keywords:** merocyanine metastable‐state photoacid, molecular photoswitches, proton transport, soft materials, solid state

## Abstract

We demonstrate here that light can be used to modulate proton transport in polymeric soft materials using a polymerizable molecular photoswitch. To this end, we design a merocyanine metastable‐state photoacid, which we use as a building block to prepare a series of light‐responsive polymers. We confirm the metastable character of the proposed monomer, and we elucidate its potential energy surface and the energies associated with its photoisomerization using quantum mechanical calculations. Interestingly, we found that when incorporated into a polymeric matrix, a photoacid loading effect impacts its photochromism and induces significant changes to the polymer nanostructure. Light stimulation of the films results in a reversible decrease in conductivity as the merocyanine simultaneously changes its net charge and functions as a photoacid by releasing protons, effecting switching as well as imparting proton conductivity to otherwise insulating polymers. We further exploit the commensurate changes to the polymer nanostructure to fabricate a light‐driven hydrogel actuator. Our work establishes a versatile synthetic platform for the design of photo‐modulated proton‐conductive systems, offering new opportunities for responsive materials and iontronics applications.

## Graphical abstract

The covalent incorporation of merocyanine metastable‐state photoacid into soft polymers imparts conductivity to otherwise insulating matrices, allowing light‐triggered control of charge and structure, offering a new platform for photo‐responsive, ion‐conducting materials.

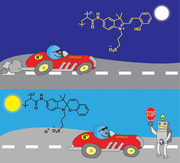

## 1 Introduction

Polymers and soft materials capable of transporting ions have received increasing attention because of their application in cutting‐edge technologies such as fuel cells [1, 2] and metal‐ion batteries [3, 4]. In these materials, ion transport is classically regulated by parameters such as temperature, local chain mobility, macroscopic mechanical properties, and the presence of small molecules such as water or low molecular weight organic moieties.

Traditionally, the ion transport in these materials is regarded as a static property at a given equilibrium condition. However, several active materials and systems present in nature, such as proton pumps or chemical synapses, require modulation of their ion transport properties upon stimuli [5, 6, 7]. To mimic this behavior, recent examples of systems and materials capable of altering their ionic conductivity using external stimuli such as light started to appear [8, 9, 10]. The possibility to remotely control the ion transport‐related properties of manmade materials opens thus the opportunity to prepare smart devices where protons or ions can be used to transport information or trigger processes, similarly to what is observed in biological systems.

Among the available external stimuli, light is a particularly appealing one since it is usually non‐destructive, can be delivered locally, and does not produce chemical waste. Once again, nature offers some extraordinary examples of systems working in this way. For instance, bacteriorhodopsin [11], a protein found in the cell membrane of a species of bacteria, which acts as a proton pump to trigger the production of ATP. Its peculiarity resides in the fact that, to trigger the pumping, energy harvested in the form of light is necessary, making it a light‐activated biological proton pump.

The current approach to mimic the behavior of biological proton pumps and, in general, systems able to modulate their ionic conductivity upon irradiation with light consists of combining two main components: a matrix and a light‐responsive unit. It has been shown that the matrix can already be a proton conductive [12] or, more commonly, a porous framework [13, 14], while the light‐responsive unit is usually an excited‐state photoacidic moiety or a molecular photoswitch. Excited state photoacids have been successfully used as additives [15], or have been covalently linked to biopolymers [16], and in both cases, photo‐modulated proton transport has been achieved. The photogenerated acidic state achieved by using these systems tends to decay quickly (in the order of ps) into their non‐acidic ground state, making the spatiotemporal modulation of the proton mobility in materials challenging to be efficiently exploited.

Light‐controlled proton transport has been demonstrated using azobenzene [17, 18] and spiropyran‐like [19, 20, 21, 22] photoswitches; however, we could only find examples of their use in MOFs, rather than as intrinsic components of an organic material. The power of these systems resides in the fact that the photoisomerization of the photochromes modifies the binding strength of guest molecules (such as 2‐methyl imidazole [21], alcohols [22] or, more commonly, water), impacting the structure of the hydrogen‐bonding pathway and, hence, the mobility of protons. Despite the success of MOF‐based systems, their crystalline, hard, and powdery nature represents a limit for device fabrication. On the contrary, we believe that the development of alternative soft plastic materials exhibiting photo‐modulated proton transport can offer multiple advantages, such as easy processability, ability to form films and membranes, and flexibility both in terms of chemical design and optical transparency. To achieve this aim, the incorporation of spiropyran‐like photoswitches in polymeric systems is very attractive [23, 24, 25, 26, 27, 28, 29, 30].

Here, we report the synthesis and characterization of a series of light‐responsive polymers that directly incorporate a photoswitch and can modulate their proton transport properties upon photoirradiation. Central to our design is the synthesis of a merocyanine metastable‐state photoacid‐based monomer [31, 32, 33] that undergoes reversible changes in electrostatic interactions and proton release under blue‐light irradiation. Incorporation of this photoacid directly into the chemical structure of the polymer matrix influences not only the polymers’ photochromic behavior but also their nanostructure and chain mobility. By extensive characterization, we demonstrate a clear relationship between monomer loading and proton conductivity, arising from the balance between percolation pathways and chain dynamics. Importantly, solid‐state photoactivation of these polymers enables reversible modulation of conductivity, driven by the dual function of the photochromic units: light‐induced proton release and redistribution of charge density, which together disrupt electrostatic interactions, enhance Coulombic repulsion, and alter polymer hydrophilicity. We further exploited this photoinduced modulation of electrostatics to fabricate light‐responsive actuators from hydrogels of our merocyanine materials.

## 2 Results and Discussion

### 2.1 Design and Synthesis of Light‐Responsive Polymers

**FIGURE 1:**
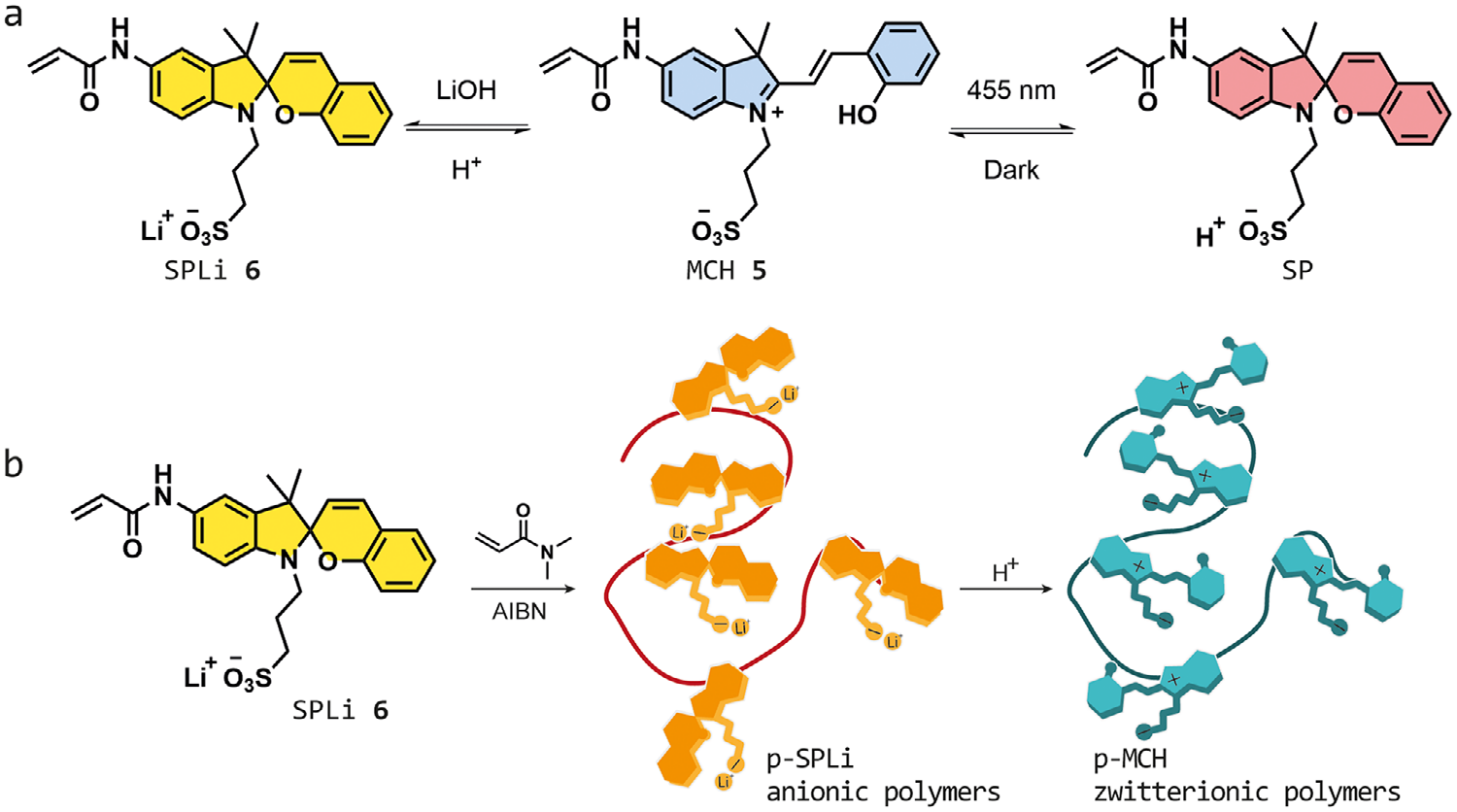
(a) Photochromism (right) and acidochromism (left) of polymerizable merocyanine metastable‐state photoacid (MCH 5). MCH‐to‐SP photoisomerization is triggered by irradiation with blue light, and MCH to SPLi ring closing is triggered by the treatment of MCH with a base, in this case LiOH. (b) Schematic synthesis of p‐MCH polymer series. SPLi (6) is used as a monomer because of its increased solubility in organic solvents, and it is co‐polymerized with N,N‐dimethyl acrylamide. The formed polymer (p‐SPLi) is treated with an acid to induce the SPLi to MCH ring opening, to form the p‐MCH polymer series.

To prepare the light‐responsive polymer, we selected merocyanine metastable state photoacid (MCH **5**, Figure 1a) as a building block. Merocyanine photoacids stand out as photoswitches as they have many interesting properties, such as sensitivity to visible light irradiation, metastability, negative photochromism, and, as there is no overlap between the main absorbance peak of MCH and its corresponding closed SP form, MCH photoswitches are also extremely efficient [34]. Additionally, when compared to other photochromes, the photoisomerization of MCH **5** to SP (Figure 1a) is associated with the change of the photochrome's net charge from zero to −1 when the proton is dissociated. All these features are pivotal for the generation of an out‐of‐equilibrium state where the proton transport properties of the bulk material can be altered using light.

To isolate the effect of MCHs switching on proton mobility in the solid state, we selected a co‐monomer that is intrinsically non‐conductive to protons. This choice ensures that any observed conductivity arises solely from the photo‐responsive units. Since proton transport often relies on hydrogen‐bond networks, we further avoided co‐monomers capable of donating hydrogen bonds. At the same time, sufficient hydrophilicity was required to enable water uptake, a prerequisite for efficient proton transport. Based on these criteria, we selected N,N‐dimethyl acrylamide (N,N‐DMA) as the co‐monomer for this study.

MCH‐like molecules are generally insoluble in organic solvents (with few exceptions [35]) and only moderately soluble in water, with maximum concentrations in the range of mm before precipitation. Such solubility is insufficient to carry out radical polymerization. To overcome this limitation, we developed an acrylate‐functionalized derivative MCH **5** (Figure 1a) that retains the light‐responsiveness of classical merocyanine photoacids [32, 33]. Building on the acidochromic properties of MCH [36], we employed lithium hydroxide to generate and isolate the spiropyran lithium salt SPLi **6** (Figure 1a,b). This design affords a monomer fully soluble in organic solvents and thus suitable for radical polymerization. Moreover, **6** is particularly suited for polymerization because the sulfonic acid lithium salt prevents the generation of phenolic radical scavengers. The resulting polymers can be readily isolated as lithium salts and subsequently converted to the zwitterionic p‐MCH form via simple treatment with a proton‐exchange resin (Figure 1b).

Following the synthetic route illustrated in Scheme S1, we thus prepared a series of four polymers containing different mol% of MCH to study the effect that MCH loading has on the photochemistry and nanostructure of the polymers, using free radical polymerization. We quantified the incorporation of SPLi **6** into the polymers by ^1^H‐NMR spectroscopy (see Section S9), and from the integration of the spectrum, the following loadings of SPLi **6** were obtained: 2.4%, 3.7%, 4.9%, and 9.5%. After proton‐exchange, these polymers are termed as p‐MCH‐1, p‐MCH‐2, p‐MCH‐3, and p‐MCH‐4.

### 2.2 Quantum Mechanical Calculations

The open‐ring MCH **5** can adopt different conformers depending on the cis/trans orientation of its three central carbon‐carbon bonds (Figure S13). Using density functional theory (DFT) at ωB97X‐D/cc‐pVDZ level [37, 38], we optimized various MCH conformers and the closed‐ring SP structure. The most stable MCH conformer (Figure S14) was selected for further analysis.

**FIGURE 2:**
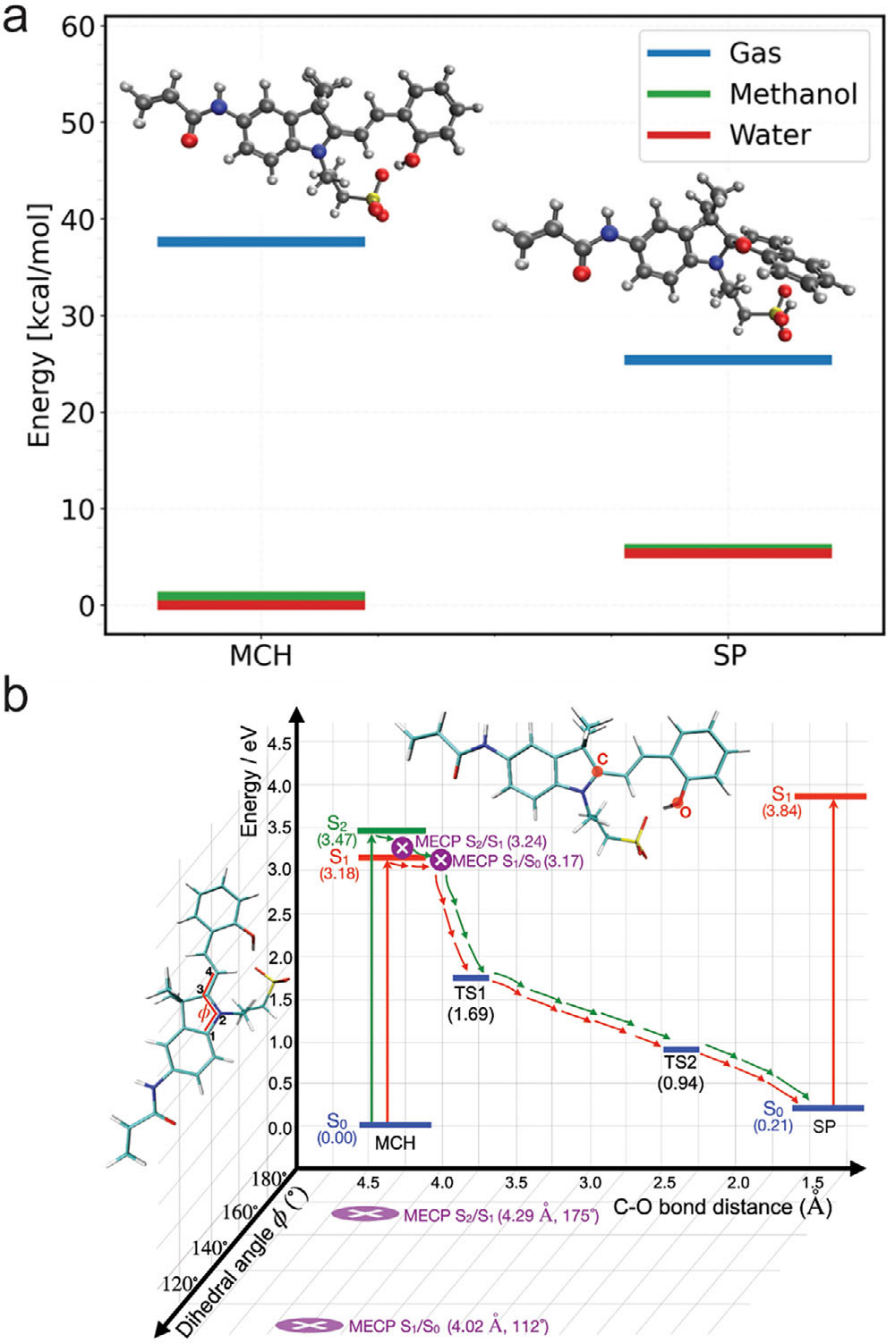
(a) Energies relative to ground state energy of an open‐ring (MCH) structure in water calculated at the ωB97X‐D/cc‐pVDZ. (b) Schematic PES in methanol along the C‐O bond distance and dihedral angle φ. Stationary points were calculated at the SF‐TDDFT B5050LYP/cc‐pVDZ level of theory. MECPs were located using the penalty function algorithm.

Calculations show that the relative stability of MCH and SP is significantly influenced by the solvent. In the gas phase, the closed‐ring SP structure is more stable than the open‐ring MCH structures (Figure 2a). However, this relative stability is reversed in the presence of a solvent modelled by the polarizable continuum model (PCM) [39, 40], with the open‐ring structure becoming more stable. This change is attributed to the solvent's stabilizing effect, which is notably stronger for the open‐ring MCH conformer compared to the more compact and less polar closed‐ring SP structure. Calculations performed in methanol and water, both polar solvents, showed that all structures were slightly more stable in water than in methanol, consistent with its higher dielectric constant and enhanced ability to stabilize charges.

To understand the ring‐closure mechanism, we performed a relaxed ground‐state potential energy surface (PES) scan along the C‐O bond formation using ωB97X‐D/cc‐pVDZ with methanol modelled by PCM. The scan revealed two barriers of 1.69 eV (38.97 kcal/mol) and 0.94 eV (21.72 kcal/mol) (TS1 and TS2, respectively; Figure 2b), indicating that photoexcitation is required to facilitate the reaction. We then analysed the lowest singlet excited states using spin‐flip time‐dependent DFT (SF‐TDDFT) [41]. The calculations of vertical excitation energies showed that the first two singlet excited states of MCH, S1 (3.18 eV or 73.33 kcal/mol) and S2 (3.47 eV or 80.02 kcal/mol), are both bright, with oscillator strengths of 1.10 and 0.13, respectively. The closed‐ring SP exhibits a higher‐energy singlet excitation at 3.84 eV (88.55 kcal/mol) with an oscillator strength of 0.78.

Finally, we explored possible relaxation pathways following photoexcitation by performing minimum energy crossing point (MECP) searches starting from the open‐ring and closed‐ring geometries. From the open‐ring conformer, both S2/S1 and S1/S0 MECPs were successfully located. The optimization, initiated from the closed‐ring geometry, however, did not converge, consistent with experimental observations that the ring‐closing process is more favorable, whereas the reverse ring‐opening reaction is generally not observed. Notably, the S1/S0 MECP involves structural distortion primarily along a dihedral coordinate, denoted φ, rather than along the C‐O bond length (Figure S15). To capture this multidimensional character, φ was included as an additional reaction coordinate in the schematic PES representation (Figure 2b).

Overall, these results indicate that excitation of MCH to S1 enables the system to reach a crossing point with the ground state at 3.17 eV, providing a nonradiative decay pathway leading to the SP product (red lines). Excitation to S2 similarly allows access to an S2/S1 MECP at 3.24 eV, followed by internal conversion to S1 and subsequent relaxation via the S1/S0 MECP (green lines). These observations reveal that photoexcitation offers an efficient route for the ring‐closing isomerization from MCH to SP. All quantum chemical calculations were performed using the Q‐Chem 6.2 software [42].

### 2.3 Polymer Photochromism

**FIGURE 3:**
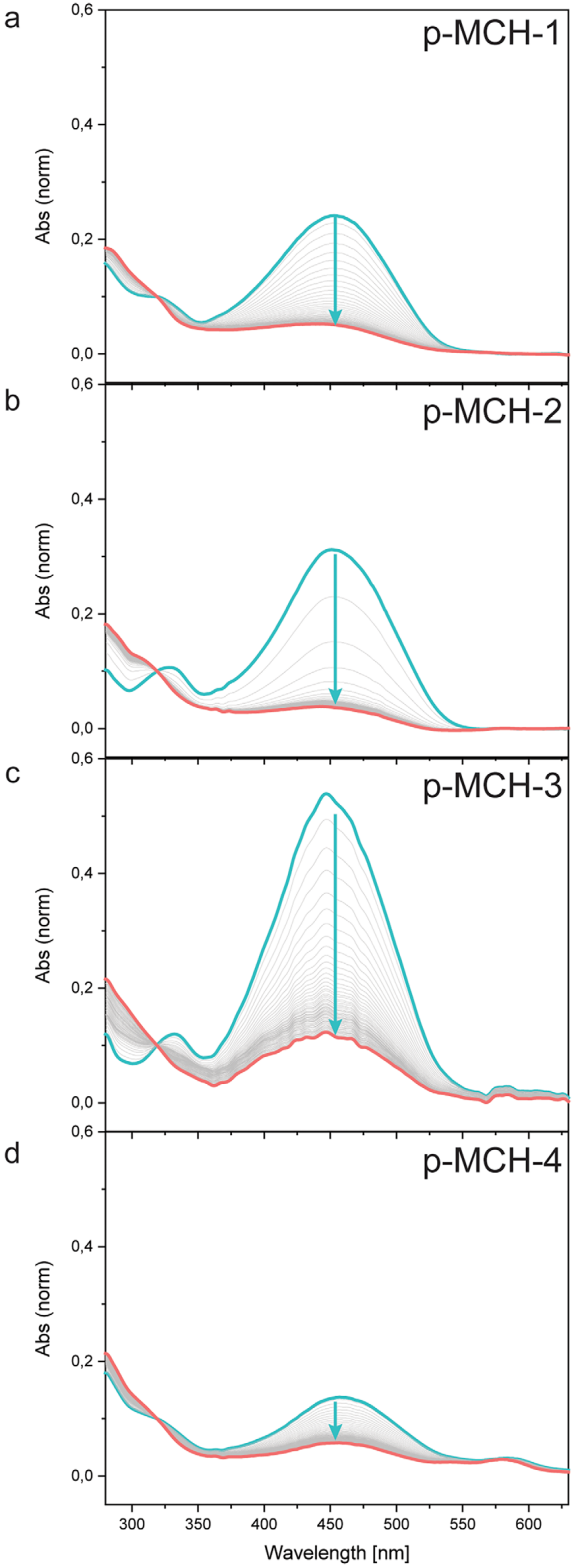
Evolution of the absorbance spectra of (a) p‐MCH‐4, (b) p‐MCH‐3, (c) p‐MCH‐2, (d) p‐MCH‐4 during irradiation with 455 nm LED in solid state. Blue line= dark spectra, salmon line= 300s of irradiation. Normalized at 319 nm.

All the prepared MCH‐decorated polymers exhibit blue‐light responsiveness in aqueous solution (Figure S3). Moreover, as we target to achieve solid‐state photochromism to alter the proton transport properties of solid state polymeric films, the light response of the polymers in the solid state was evaluated by UV–vis absorption spectroscopy on drop‐cast films. As shown in Figure 3a–d, all p‐MCH polymers display the characteristic MCH absorption band centred at 455 nm, which decreases in intensity under blue‐light irradiation, indicating that the photochromic properties of **5** are not lost upon incorporation in the polymeric chain, and in the solid state.

**FIGURE 4:**
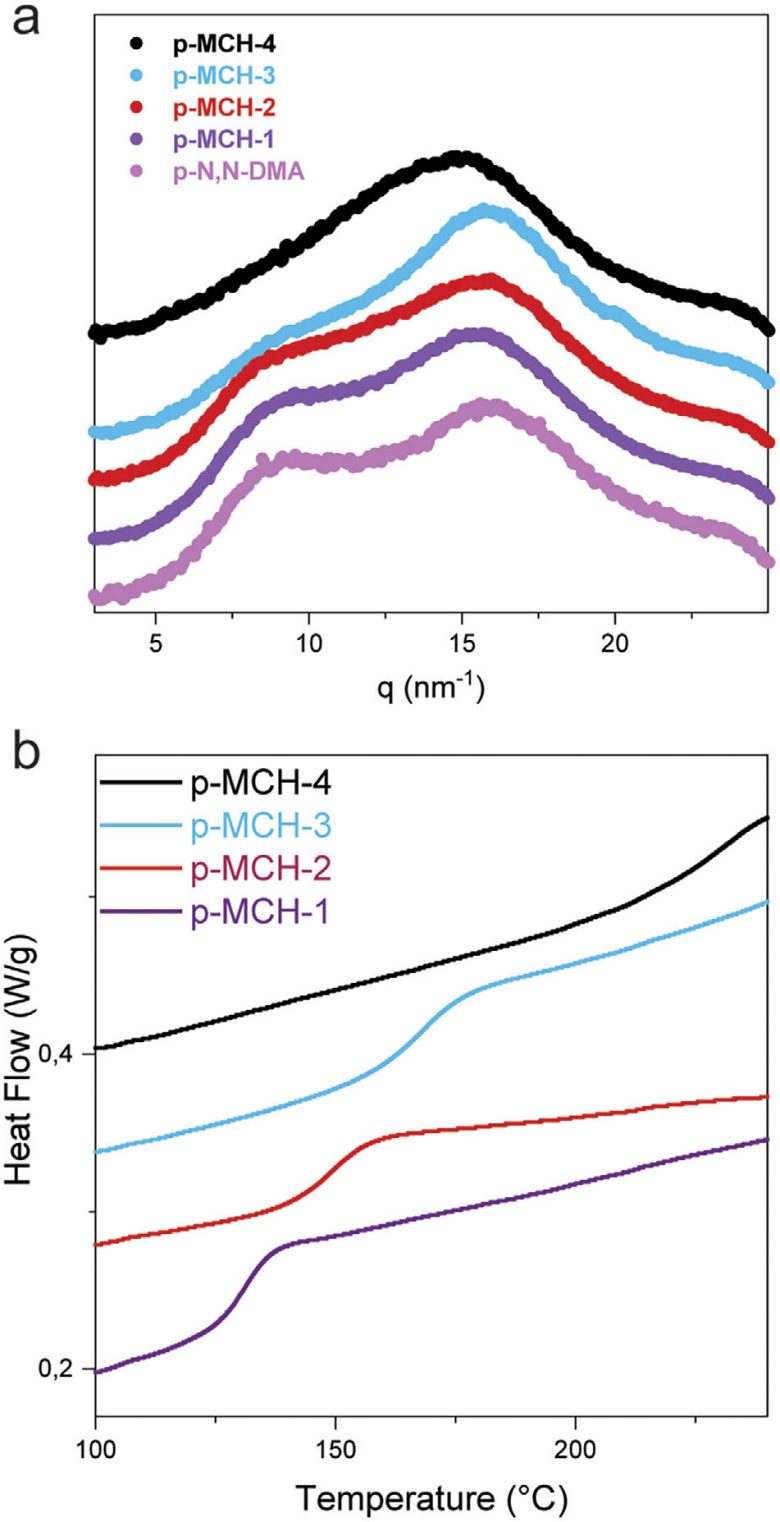
(a) GIWAXS profiles of p‐MCH polymers. Increasing the MCH moiety content in the polymeric chain led to the disappearance of the peak at q=8 nm^−1^, associated with long‐range order. (b) Zoom of the DSC profiles of the p‐MCH polymers, where the increase of Tg is associated with an increase of MCH monomer content.

Nonetheless, the system presents some peculiarity as none of the samples show complete disappearance of the absorption peak upon irradiation, in contrast to what was observed in solution. These results indicate that, although solid state isomerization is achieved with the present designs, spatial confinement in the polymer films reduces the switching efficiency of the photoactive units [43, 44]. Moreover, from Figure 3a–d, it is clear how the loading of monomer **6** into the polymeric chain has an effect on the intensity of the MCH peak, centred at 455 nm. In fact, it is clear from the UV–vis investigation of the full polymer series that an increased loading of polymerizable merocyanine photoacid **5** into the polymer initially translates into an increase in intensity of the MCH peak (for p‐MCH1, 2, 3, Figure 3a–c) and further incorporation of photoactive monomer results in the quenching of its absorption peak. The observed decrease in peak intensity for p‐MCH‐4 correlates with the loss of long‐range order in the solid state, also observed in X‐ray scattering experiments (Figure 4a). This suggests that, at high loading, MCHs associate in a locally correlated but globally disordered manner, possibly through intermolecular coulombic interaction. The increased MCH content enhances short‐range electrostatic and electronic interactions between zwitterionic units, disrupting the polymer's overall packing and long‐range organization. Such assemblies cause the distortion of the molecular geometry and hinder coherent chromophore alignment. These distortions reduce the effective overlap of electronic transitions between neighbouring chromophores [45, 46, 47], leading to a hypochromic effect [48, 49, 50], and as a result, the intensity of the merocyanine absorption band is diminished.

### 2.4 Structure‐Properties Relationship

To assess the effect of MCH loading on polymer nanostructure, we performed Grazing‐Incidence Wide‐Angle X‐ray Scattering (GIWAXS) on cast p‐MCH films. As shown in Figure 4a, the GIWAXS profile of pure poly(N,N‐DMA) is characterized by the presence of two broad peaks at ∼8 and ∼17 nm^−1^. The higher‐q peak is attributed to short‐range order within the polymer, reflecting orientational or conformational correlations between adjacent chain segments, despite the absence of semi‐crystallinity. Conversely, the low‐q peak is associated with the polymers long‐range order, reflecting positional and orientational correlations of polymer chains over extended distances [51]. As shown in Figure 4a, increasing the MCH content progressively disrupts this long‐range order, yielding more disordered materials, while short‐range order remains mostly unaffected. We attribute this loss of order to the proximity of MCH units, which promotes supramolecular electrostatic interactions between charged monomers, introducing chain distortion and causing structural heterogeneity and disorder.

Increasing the concentration of MCH side groups also affects the thermal relaxation of the polymer chains. Differential scanning calorimetry (DSC) reveals a systematic increase in the glass transition temperature (Tg) with increasing MCH loading (Figure 4b). For the pure polymer, Tg lies at 115–118°C [52, 53], whereas for p‐MCH‐4 it rises to ∼230°C, approaching the onset of thermal decomposition (Figure S7). These results indicate that MCH side groups not only disrupt long‐range order but also restrict chain relaxation, such that progressively higher energy is required to achieve the transition from the glassy to the rubbery state. These observations further suggest that the quenching of the MCH absorption peak observed in the solid‐state UV–vis spectra (Figure 3a–d) is caused by the high loading of the MCH moiety, which is in turn directly related to a drastic increase in the rigidity of the polymer chains and a disruption of the polymer nanostructure.

### 2.5 Photo Controlled Proton Mobility

**FIGURE 5:**
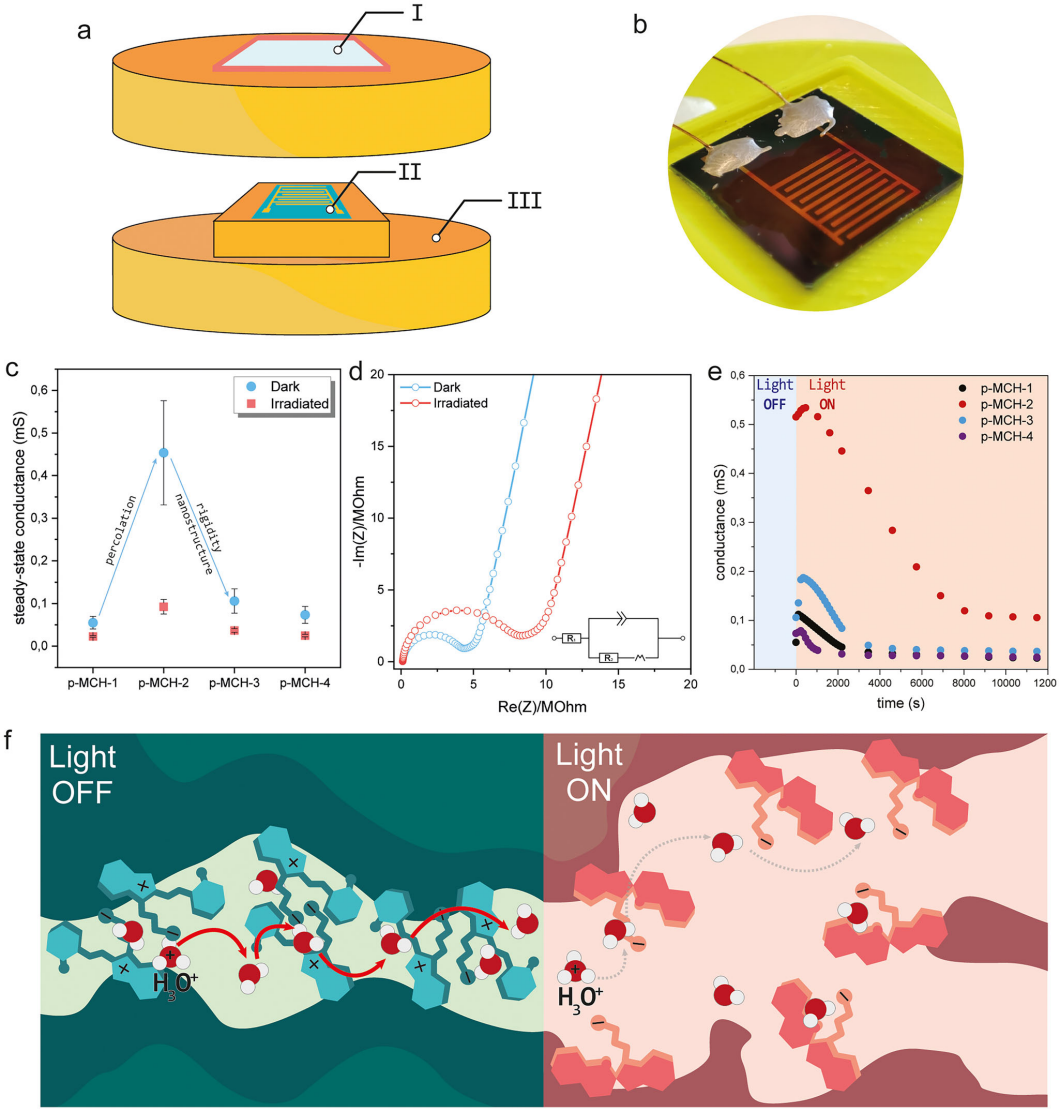
(a) Scheme of the used setup for irradiation of the p‐MCH polymer series during EIS measurements. I) Glass window to ensure light penetration, II) Interdigitated electrode with thin polymer film casted on top, III) compartment for wet cotton wool to ensure RH=100%. (b) Picture of an interdigitated electrode with a thin film of p‐MCH‐2 casted on top. (c) Steady‐state conductance of p‐MCH polymer series in the dark and after irradiation with light. Error bars represent the standard deviation derived from three independent experiments using three separate samples for each polymer derivative. (d) Nyquist plots and equivalent circuit for p‐MCH‐2 in the dark and after irradiation. (e) Evolution of the conductance of p‐MCH polymer series upon irradiation. There is a clear initial increase in conductance, followed by a slow decrease caused by the change of the physicochemical properties of the material, such as percolation, coulombic interactions, and hydrophilicity. The relative error for the conductance values obtained from the equivalent circuit fitting of the EIS data was less than 3%. (f) Schematic representation of the Grotthus mechanism taking place in the p‐MCH films in the dark, where attractive coulombic interactions ensure efficient proton hopping and percolation (left), and upon irradiation, where repulsive electrostatic interactions destroy the percolation of the materials.

Next, we evaluated the proton conductivity of the p‐MCH polymer series using electrochemical impedance spectroscopy (EIS). Since the parent pN,N‐DMA backbone is insulating, any observable proton transport is related to the inclusion of MCH. Solid‐state UV–vis measurements on hydrated films (Figure 3a–d) indicate that the phenol group of the MCH moiety remains largely undissociated, as in all dark‐state spectra, the absorption band corresponding to the phenolate (∼550 nm in water for **5**; Figure S2a) is absent. For this reason, we associate the mobility of protons inside the polymeric films with the presence of zwitterionic units. It has been previously shown that the presence of zwitterionic guests [54] or zwitterionic units [55] in MOFs greatly increases their proton transport properties, thanks to the hydrogen bonding interactions that zwitterions have with water [56] as they can serve as proton hopping domains and help water dissociation [57]. In the dark, the polymer lacks strongly acidic groups, so protons are supplied primarily by the self‐dissociation of water. Zwitterionic MCH units mediate proton hopping, and proton concentration is expected to scale linearly with the mol% of MCH on the polymer chain (Equation 1). Interestingly, we observe a nonlinear relationship between MCH content and steady‐state proton conductivity in the dark (Figure 5c). While increasing the MCH fraction from p‐MCH‐1 to p‐MCH‐2 enhances conductivity, further increases beyond ∼4 mol% result in a decrease, demonstrating that higher MCH loading adversely affects proton transport.

(1) $$\sigma^{H+}=F\left[H^{+}\right]\mu \prime_{H+}$$

Equation (1) Proton conductivity. F = Faraday constant, [H+] = proton concentration, µ′_H+_ = effective proton mobility.

This behavior can be explained by considering two different phenomena occurring in the materials. On one hand, the one order of magnitude of the increase in conductance observed when increasing the MCH content from 2.4% in p‐MCH‐1 to 3.7% in p‐MCH‐2 cannot be simply attributed to the increased number of protons according to Equation (1). We rather attribute this sudden increase in conductance to the establishment of an efficient zwitterionic percolation path for the protons. Proton transport can only occur above the percolation threshold, which in this case is directly related to the density of MCH units. Above a critical MCH content, there are sufficient sites, evenly distributed, to enable proton transport. However, GIWAXS and DSC data show a loss of long‐range order and increased chain rigidity at very high MCH densities (Figure 5c). Thus, proton conductivity increases with increasing MCH density until the polymer becomes too disordered to sustain efficient proton transport.

Owing to the light‐switching properties of the MCH side units, we have further tested the photo modulation of the proton transport in our materials. Figure 5 shows the change in conductance under light irradiation. At steady state conditions (i.e., under continuous light irradiation and when the EIS spectra do not change anymore with time), the system conductance decreases for all samples. The trend in the steady state conductance under illumination closely resembles the one observed in the dark, with the p‐MCH‐2 polymer (3.7% of MCH units) showing the lowest resistance. This observed decrease in conductance is intriguing, since exposure to light generates a strongly acidic sulfonic group that, in the presence of water, is fully dissociated and provides readily protons. To shine more light onto the photo modulation mechanism of proton transport, we conducted time‐resolved EIS measurements, following the change of resistance (i.e., conductance) during illumination until the steady state value is reached. All the studied p‐MCH polymers show a non‐monotonic change of conductance with time, where a first rapid increase is followed by a slower decrease in conductance with time (Figure 5e). We attribute the initial increase in conductance to the release of acidic protons in the systems, caused by the MCH to SP photoisomerization.

The SP form increases proton concentration through the terminal sulfonate group because of the very low pKa of the conjugate sulfonic acid, which, according to Equation (1), increases the concentration of free protons in the systems. This hypothesis is supported by the fact that the solid‐state photoisomerization kinetics from p‐MCH to p‐SP are in good agreement with the timescale for this initial increase in conductance (Figure S6). The MCH‐to‐SP photoisomerization leads to the formation of an acidic moiety, nominally increasing the proton concentration in the material. However, for longer time scales, the conductance slowly decreases. We attribute this second phenomenon to the fact that the photoisomerization of the MCH units in the polymers causes the net charge of the responsive units to change from 0 to −1. This transformation profoundly alters supramolecular coulombic interactions, leading to increased structural disorder (as evidenced by X‐ray data, Figure 4a) and a drastic reduction in effective proton mobility. The irradiation‐induced disordering in the polymer nanostructure results in a distortion of the proton‐conductive domains, possibly impacting the percolation of the materials, and negatively impacting their conductance (Figure 5f, right side). On the contrary, when the polymers are in the dark, favorable coulombic interactions are taking place between zwitterionic units [58, 59, 60], which contribute toward a better‐defined proton‐conducting path and hence a higher proton conductivity [54, 55] (Figure 5f, left side).

It is interesting to note that the decrease in proton conductivity at the longer time scales is more pronounced for the p‐MCH‐2, indicating that the effect of proton‐conducting path distortion and destruction has a larger impact on the proton transport properties of the best‐performing sample. Since the p‐MCH‐2 polymer presents an optimum balance between percolated structure, chain ordering, and not too high chain rigidity, it is the one experiencing the more dramatic impact of the photo‐modulation‐induced distortion, which also means the biggest extent of photo‐modulated transport change. To the best of our knowledge, this behavior has never been reported before and seems to be unique for soft polymeric materials. Previously reported systems based on metal organic frameworks (MOFs) as a scaffold and photoswitches either covalently linked to them [22], or used as dopants [19, 20, 21] do not show such a non‐monotonic increase in proton mobility while irradiated.

**FIGURE 6:**
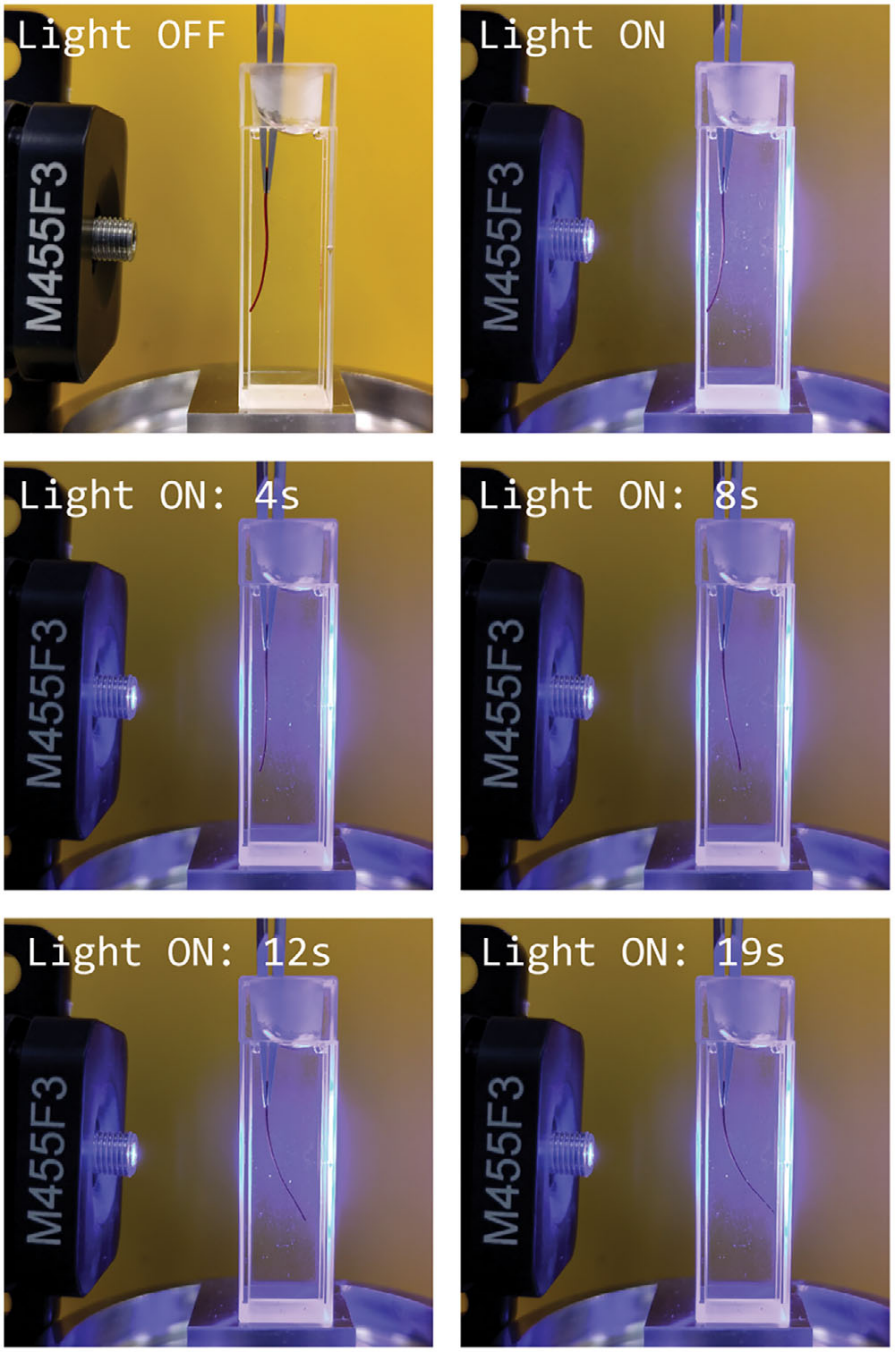
Light‐induced negative phototaxis of thin hydrogel, caused by the MCH‐to‐SP photoisomerization. The hydrogel bends away from the light source, confirming that the change in hydrophilicity and in the electrostatic interactions within MCH monomer causes the change of the physicochemical properties of the polymers, and consequently their proton transport properties.

The change in the electrostatic interactions that cause structural distortions and conductance decrease can be visualized and exploited also at the macroscopic level to prepare light‐triggered actuators. To this end, we prepared a hydrogel having the same composition as the p‐MCH polymers series, which, upon irradiation with visible light, bends away from the light source within a few seconds (Video S1), showing negative phototaxis. Upon irradiation, the MCH‐to‐SP isomerization takes place, then the single monomers are subjected to repulsive interactions among each other and, concomitantly, the increased hydrophilicity of the system allows local swelling of the gel (Figure 6). Since the hydrogel is irradiated only from one side, the degree of conversion from the MCH‐to‐SP form decreases across the thickness and results in a non‐homogeneous swelling, higher toward the light. It is interesting to notice that the MCH‐to‐SP photoisomerization involves not only a change in net charge from 0 to −1, but also a structural transformation from a planar, conjugated geometry to a 3D, unconjugated spirocyclic form (Figure 1a). However, we propose that this conformational change is not the primary driving force for hydrogel expansion. Previous studies have shown that systems undergoing similar conformational transitions can exhibit either positive or negative phototaxis, with the directionality determined by the net charge of the photogenerated metastable isomer rather than the structural change itself [61, 62, 63]. The combination of changes in hydrophilicity, non‐homogeneous swelling, and electrostatic interactions results in a bending‐away‐from‐the‐light actuation mode of the hydrogel. These same phenomena, being active at the nanoscale, are also responsible for the photo modulation of ion (proton in this case) transport, enabling light‐controlled ionic transport.

## 3 Conclusions

We present here the synthesis of a series of polymers exhibiting solid state photochromism that are based on a merocyanine metastable‐state photoacid monomer. Together with the addition of an acrylamide co‐monomer, random photo‐responsive copolymers can be obtained. The addition of a comonomer does not alter the photochromic properties of the photoacid. Quantum mechanical calculations show that solvent polarity significantly affects the stability of SP and merocyanine MCH, and a solvent stabilization effect was found. The potential energy surface analysis revealed key crossing points that enable efficient non‐radiative photoisomerization, explaining the reversible switching observed experimentally.

Through thermal and X‐ray analysis, we demonstrated that the incorporation of the MCH side units has a profound impact on the polymer structure and dynamics, resulting in increased chain rigidity and loss of long‐range order upon introduction of the light‐responsive units, where both effects can be attributed to the electrostatic zwitterionic interactions between MCH units.

We then demonstrated that MCH units can induce proton conductivity in otherwise resistive polymers, and that the proton conductance in the dark strongly depends on MCH content. We unravel how the optimal content is dictated by a balance between percolation and mobility: sufficient MCH is required to enable effective percolation and facilitate proton transport, but excessive loading leads to increased chain rigidity and a loss of nanostructure, ultimately reducing conductivity. We found that p‐MCH‐2, containing 3.7 mol% MCH, provides the best trade‐off between these competing factors, exhibiting the highest proton conductivity in the dark.

Upon light irradiation, the MCH units are converted into charged, acidic spiropyran (SP) forms, bearing a sulfonic acid group at their termini, which alters the properties of the photo‐responsive polymers and was exploited in this work to change the proton transport properties of the materials. All polymers showed light‐modulated proton transport with an intriguing time‐dependent behavior, featuring a quick conductance increase, followed by a slow decrease over time until a steady state is reached. We attribute this behavior to the dual nature of the MCH photoswitch that, upon isomerization, acts as a photoacid, releasing protons and increasing conductivity, while simultaneously undergoing a charge shift from zwitterionic to anionic, which alters monomer–monomer interactions from attractive to repulsive and changes the polymer's hydrophilicity. The extent of proton transport photo‐modulation is again dependent on the MCH content, with the 3.7 mol% MCH polymer exhibiting the larger difference in conductance between dark and light illumination.

Finally, we make use of this peculiar change in electrostatic interactions and change in hydrophilicity to create a photo‐responsive hydrogel that can be light‐actuated. This actuation is at the second time scale, and it arises from a combination of altered intermonomer electrostatic interactions and increased water affinity of the negatively charged light‐responsive units.

Overall, our study provides valuable insights into the structure‐property relationships of soft MCH‐based light‐responsive polymers and their potential for applications in dynamic materials. Our findings underscore the importance of optimizing polymer composition, nanostructure, and electrostatic interactions to achieve robust photo‐switching behavior, light‐modulated proton conductivity, and macroscopic actuation, positioning these systems as promising candidates for future responsive materials and iontronics devices.

## Conflicts of Interest

The authors declare no conflicts of interest.

## Supporting information




**Supporting File**: smll72898‐sup‐0001‐SuppMat.docx.

**Supporting File**: smll72898‐sup‐0002‐VideoS1.zip.

## Data Availability

The data that support the findings of this study are available from the corresponding author upon reasonable request.
