# Supplementary material for: Photo‐Modulated Proton Transport in Merocyanine Metastable‐State Photoacid Based Polymers
Source: Small. 2026 Feb 25;22(23):e14786. doi: 10.1002/smll.202514786 (PMC13100567; doi:10.1002/smll.202514786)
Supplement: Supplementary file 1 — Supporting File: smll72898‐sup‐0001‐SuppMat.docx. [file SMLL-22-e14786-s002.docx]

Supporting Information for the paper:

Photo-modulated proton transport in merocyanine metastable-state photoacid based polymers

*Gianni Pacella, Mira Kim, Rachael Hannah, Ryan C. Chiechi, Shirin Faraji, Giuseppe Portale**

1- General remarks

2- Synthesis

2.1.- Monomer synthesis

2.2.- Polymers synthesis

2.3.-Hydrogel synthesis

3- UV-Vis absorbance spectroscopy studies

3.1.- UV-Vis of monomer

3.2.- UV-Vis of polymers in solution

3.3.- UV-Vis of polymers in the solid state

4- Polymers characterization

4.1.- Size exclusion chromatography (GPC)

4.2.- Thermo Gravimetric Analysis (TGA)

5- Electrochemical Impedance Spectroscopy

5.1.- EIS with in situ irradiation

5.2.- Arrhenius analysis

6- Irradiation experiments on light-responsive hydrogel

7- Grazing Incident Wide Angle X-ray Scattering (GIWAXS)

8- Quantum mechanical calculations

9- NMR spectra

10- References

1-General remarks

All reagents and solvents have been purchased from Sigma Aldrich, TCI Europe, Acros Organics, BOOM Chemicals, and were used without further purifications unless specified. Dry solvents have been obtained from an MBraun SPS-800 solvent purification system. All the reactions have been carried out under argon atmosphere. Flash column chromatography was carried out using silica gel Davisil LC60A (Merck type 9385, 230400 mesh) using the specified eluents.

Standard NMR spectra (^1^H and ^13^C) have been recorded on a Varian Mercury-Plus (400 MHz) spectrometer at 298 K (25 °C).

High-resolution spectroscopy (HRMS) was performed on an LTQ Orbitrap XL spectrometer with electrospray ionization (ESI) as the ionization technique.

UV-Vis absorption spectra were recorded on an Agilent 8453 UV-Vis Diode Array System, equipped with a Quantum Northwest Peltier controller in 10 mm quartz cuvettes. Irradiation experiments were performed using LEDs from Thorlabs Incorporated (455 nm; 1 A).

Electrochemical impedance spectroscopy (EIS) measurements have been performed similarly to what has already been previously reported by our group.^[1]^ EIS measurements were performed using an SP-300 Biologic impedance spectrometer. The oscillating amplitude was fixed at 100 mV, and impedance spectra were collected by scanning the frequency from 7 MHz to 100 MHz in the logarithmic scale, with 10 points per decade. The samples were cast on gold interdigitated electrodes (IDEs) deposited on silicon substrates with a 1000 nm SiO_2_ insulating top layer and placed in a humidified chamber with relative humidity of 100%. The impedance data were fitted using the EC-Lab Zfit software.

Thermogravimetric analysis (TGA) was performed on a Perkin Elmer STA 6000 instrument under a continuous nitrogen flow (30 ml/min). Samples have been heated from 30 to 700 °C at a heating rate of 10 °C/min.

Differential scanning calorimetry (DSC) measurements were conducted on a TA Instruments DSC Q1000 under nitrogen atmosphere. The samples were heated to 100 °C and cooled back to −90 °C at a rate of 10 °C/min. The second heating cycle was then used for analysis.

Gel Permeation Chromatography (GPC) was performed in DMF containing 0.01 M LiBr on a Viscotek GPCmax equipped with model 302 TDA detectors and two columns (Agilent Technologies-PolarGel-L and M, 8 µm 30 cm) at a flow rate of 1.0 ml/min and 50 °C. PMMA with narrow polydispersity (Polymer Laboratories) was used as a standard to construct a universal calibration curve.

GIWAXS measurements were performed using the Multipurpose Instrument for Nanostructure Analysis (MINA) X-ray scattering instrument built on a Cu rotating anode source (λ = 1.5413 Å). 2D patterns were collected using a Vantec 500 detector (2048 × 2048 pixel array with pixel size 68 × 68 μm) located 93 mm from the sample. The polymeric films were placed in reflection geometry and measured using an incident angle αi = 0.2° between the sample surface and the incoming beam set by means of a Huber goniometer.

2-Synthesis


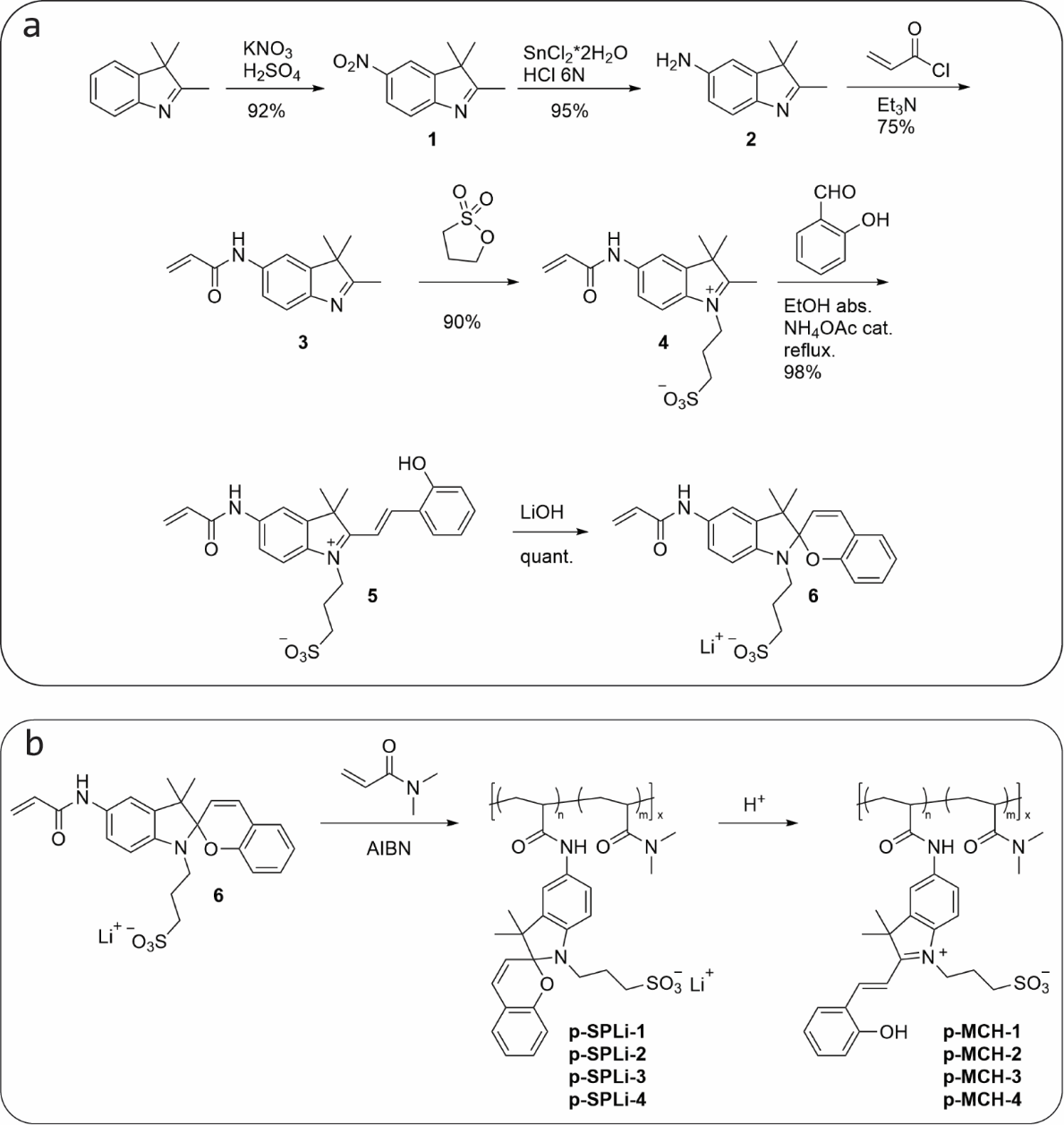


Scheme S1. a) Steps towards the synthesis of SPLi monomer 6. b) Synthesis of p-MCH-n polymers

2.1.-Monomer synthesis

# 2,3,3-trimethyl-5-nitro-3H-indole (1):

2,3,3-trimethylindolenine (6.3 ml, 40 mmol) was added to concentrated sulfuric acid (40 ml) at 0 °C, under argon atmosphere. Then, a cold solution of KNO_3_ (5.26 g, 52 mmol) in concentrated sulfuric acid (27 ml) was slowly added to the mixture. The reaction was checked by TLC (1:1 EtOAc:Hex). After 24 hours, the mixture was treated with NaOH 5N until alkaline pH, then extracted with dichloromethane (3x200 ml), and dried over dry Na_2_SO_4_. The solvent is then removed under reduced pressure. The title compound was isolated as a red solid (7.42 g, 92% yield).

^1^H NMR (400 MHz, CDCl_3_) δ: 8.23- 8.21 (1H, dd), 8.13 (1H, d), 7.59-7.57 (1H, d), 2.33 (3H, s), 1.34 (6H, s).

^13^C NMR (75 MHz, CDCl_3_) δ: 194.24, 158.79, 146.64, 124.55, 120.02, 117.17, 54.50, 22.72, 15.97.

MS-ESI (m/z): [M+H]^+^ calc. for C_11_H_13_N_2_O_2_ 205.0972; found [M+H]^+^ 205.0968

# 2,3,3-trimethyl-3H-indol-5-amine (2):

Compound 1 (7.04 g, 34.47 mmol) was added to a hydrochloric acid solution in water (200 ml, 6N) and stirred until complete dissolution. SnCl_2_*2H_2_O (42.60 g, 188.79 mmol) was then added to the mixture that was stirred under reflux for 17 hours. The reaction was checked by TLC (SiO_2_, EtOAc:Hex = 6:2). The mixture was then treated with NaOH 5N until alkaline pH, then extracted with CH_2_Cl_2_ (3x100 ml), BRINE (1x100 ml), and dried over anhydrous Na_2_SO_4_. The volatiles were removed under vacuum to yield the title compound as a yellow/light brown solid (5.66 g, 95% yield).

^1^H NMR (400 MHz, CDCl_3_) δ 7.29 (1H,d), 6.61- 6.56 (2H, m), 3.65 (2H, s), 2.20 (3H, s), 1.24(6H, s).

^13^C NMR (101 MHz, CDCl_3_) δ 184.18, 147.26, 145.84, 144.33, 120.11, 113.83, 108.92, 53.35, 23.43, 23.24, 15.14.

MS-ESI (m/z): [M+H]^+^ calc. for C_11_H_15_N_2_ 175.1230; found [M+H]^+^ 175.1228

# N-(2,3,3-trimethyl-3H-indol-5-yl)acrylamide (3):

Compound 2 (2.31g, 13.27 mmol) was dissolved in 26 ml of dry CH_2_Cl_2_ under inert atmosphere of argon. The solution was then cooled down to 0 °C, and triethylamine (2 ml, 14.35 mmol) was added. Acryloyl chloride (1.2 ml, 14.76 mmol) in dry CH_2_Cl_2_ was slowly dropped into the solution over 20 minutes. The reaction was left under stirring for 18 hours. Subsequently, the organics were extracted with a sodium bicarbonate saturated solution (100 ml), distilled water (3x100 mL), and the organic phase was dried over anhydrous Na_2_SO_4_. After removing the solvent in vacuum, the crude product was purified by flash column chromatography (SiO_2_, EtOAc:Hex = 85:15) (R_f_=0.34) to yield the title compound as an orange/yellow sticky solid (2.26 g, 75% yield).

^1^H NMR (400 MHz, CDCl_3_) δ 8.50 (1H, s), 7.94 (1H, s), 7.39 (1H, d), 7.23-7.20 (1H, dd), 6.44-6.40 (1H, dd), 6.35-6.28 (1H, dd), 5.71- 5.68 (1H, dd), 2.23 (3H, s), 1.25 (6H, s).

^13^C NMR (101 MHz, CDCl_3_) δ 187.98, 164.08, 149.77, 146.49, 136.02, 131.32, 127.41, 119.58, 119.31, 114.17, 53.91, 22.96, 15.29.

MS-ESI (m/z): [M+H]^+^ calc. for C_14_H_17_N_2_O 229.1335; found [M+H]^+^ 229.1332

# 3-(5-acrylamido-2,3,3-trimethyl-3H-indol-1-ium-1-yl)propane-1-sulfonate (4):

Compound 3 (1.98 g, 8.67 mmol) was dissolved in dry acetonitrile (8 ml), then 1,3-propane sultone was added to the mixture. The solution was heated to reflux for 15 hours. Subsequently, the volatiles were removed in vacuum, and the remaining crude solid was filtered over a glass filter porosity 3, washed with acetone (3x50 ml), and diethyl ether (3x50 ml). The obtained powder was dried in a vacuum oven at 75 °C. After drying, the product was collected as a beige powder (2.76 g, 90% yield).

^1^H NMR (400 MHz, CD_3_OD) δ 8.24 (1H, d), 7.95 (1H, d), 7.70(1H, dd), 6.45-6.43 (2H, m), 5.83 (1H, dd), 4.71(2H, t), 3.00 (2H, t), 2.40-2.32 (2H, m), 1.60 (6H, s).

^1^H NMR (400 MHz, D_2_O+LiCl) δ 7.82 (1H, s), 7.67 (1H, d), 7.52 (1H, d), 6.35-6.20 (2H, m), 5.78 (1H, d), 4.50 (2H, t), 2.98 (2H, t), 2.27-2.23 (2H, m), 1.44 (6H, s).

^13^C NMR (101 MHz, D_2_O+LiCl) δ 196.31, 166.32, 142.85, 138.91, 137.07, 130.05, 129.16, 121.18, 115.57, 115.51, 54.58, 47.36, 46.34, 22.80, 21.76.

MS-ESI (m/z): [M+H]^+^ calc. for C_17_H_23_N_2_O_4_S 351.1373; found [M+H]^+^ 351.1366

# (E)-3-(5-acrylamido-2-(2-hydroxystyryl)-3,3-dimethyl-3H-indol-1-ium-1-yl)propane-1-sulfonate (5):

Compound 4 (10,21g, 29,13 mmol), ammonium acetate (0,45g, 5,83 mmol), and salicylaldehyde (3,65 ml, 34,96 mmol) were dissolved in absolute ethanol. The mixture was heated to reflux under inert atmosphere of argon for 17 hours. After this time, the mixture is then allowed to cool down to room temperature and filtered over a glass filter porosity 3. The red solid obtained in this way is dried in the vacuum oven at 75 °C. After drying, the product was collected as a bright red powder (12,96 g, 28,52 mmol, 98% yield).

^1^H NMR (300 MHz, DMSO-d6) δ 10.97 (1H, s), 10.58 (1H, s), 8.56-8.51 (1H, d), 8.26-8.20 (2H, m), 7.97 (1H, d), 7.85-7.79 (1H, d), 7.71 (1H, d), 7.45 (1H, m), 7.08-6.91 (2H, m), 6.51- 6.42 (1H, m), 6.31 (1H, d), 5.82 (1H, d), 4.75 (2H, m), 2.64 (2H, m), 2.17 (2H, m), 1.74 (6H, s).

MS-ESI (m/z): [M+H]^+^ calc. for C_24_H_27_N_2_O_5_S 455.1635; found [M+H]^+^ 455.1620

# Lithium 3-(5'-acrylamido-3',3'-dimethylspiro[chromene-2,2'-indolin]-1'-yl)propane-1-sulfonate (6):

Compound 5 (12.74 g, 28.04 mmol) was dissolved in 350 ml of methanol. LiOH (0.74 g, 30.84 mmol) is then added to the solution, which is allowed to stir for 20 minutes. 350 ml of DCM are then added to the solution that is subsequently filtered over a glass filter porosity 3. The volatiles are then removed under reduced pressure to afford the title compound as a purple solid (12.85g, 99% yield).

^1^H NMR (400 MHz, DMSO-d6) δ 9.91 (1H, s), 7.42 (1H, d), 7.31-7.29 (1H, dd), 7.14-7.12 (1H, m), 7.08- 7.04 (1H, m), 6.97- 6.94 (1H, m), 6.82- 6.78 (1H, m), 6.62 (1H, d), 6.58 (1H, d), 6.43- 6.36 (1H, dd), 6.20-6.16 (1H, dd), 5.71 (1H, d), 5.67- 5.64 (1H, dd), 3.18-3.11 (2H, m), 2.45-2.37 (2H, m), 1.87-1.80 (2H, m), 1.15 (3H, s), 1,07 (3H, s).

^13^C NMR (101 MHz, DMSO-d6) δ 162.88, 154.27, 144.26, 136.66, 132.67, 131.23, 130.17, 129.62, 127.39, 126.13, 120.58, 120.03, 119.41, 118.93, 114.86, 106.53, 104.94, 56.47, 52.33, 49.66, 43.00, 26.08, 25.46, 20.16, 19.00.

MS-ESI (m/z): [M+H]^+^ calc. for C_24_H_26_LiN_2_O_5_S 461.1717; found [M+H]^+^ 461.1708

2.2.-Polymers synthesis

# General procedure for the p-SPLi polymers:

Monomer 6 (2.5 eq for p-SPLi-1, 5 eq for p-SPLi-2, 10 eq for p-SPLi-3, 20 eq for p-SPLi-4) is placed in an oven dried Schlenk tube under argon atmosphere. Then, recrystallized AIBN (1eq) is added, followed by dry DMF and N,N-DMA (97.5 eq for p-SPLi-1, 95 eq for p-SPLi-2, 90 eq for p-SPLi-3, 80 eq for p-SPLi-4). Argon is bubbled into the solution for 30 minutes. Then the solution is placed into an oil bath at 75 °C and left stirring for 17-18 hours. The solution is then allowed to cool down to room temperature before slowly dropping it into diethyl ether (400 ml) under vigorous stirring. The resulting solid is then vacuum filtered over a glass filter por. 3 and washed with more diethyl ether (2x50 ml), before being dried in a vacuum oven at 75 °C overnight. After drying, the polymer presented itself as a light purple powder.

p-SPLi-4 has then been subjected to dialysis against methanol for five days, changing the methanol every two days, to remove the unreacted monomer. A 1 KDa dialysis membrane has been used. After this time, the sample has been removed from the dialysis membrane, and the solvent removed at reduced pressure to yield p-SPLi-4.

Table S1: conditions for the preparation of p-SPLi-n

| Polymer | mmol 6 | g 6 | ml DMF | Isolated yield | |
| --- | --- | --- | --- | --- | --- |
| p-SPLi-1 | 0.22 | 0.10 | 16 | 0.80g | 85% |
| p-SPLi-2 | 0.43 | 0.2 | 17 | 0.73 | 71% |
| p-SPLi-3 | 0.76 | 0.35 | 16 | 1.07 | quant |
| p-SPLi-4 | 0.76 | 0.35 | 8 | 0.28 | 43% |

# Synthesis of poly N,N-Dimethyl acrylamide (p-N,N-DMA):

N,N-DMA (2 ml, 19.40 mmol) are placed in an oven dried Schlenk tube with recrystallized AIBN (0.31g, 0.19 mmol) and dry DMF (8 ml). The solution is bubbled with argon for 30 minutes before being placed in an oil bath at 75 °C for 17-18 hours. Passed this time, the solution is cooled down to room temperature and slowly dropped into 400 ml of diethyl ether under vigorous stirring. The resulting white solid is vacuum filtered over a glass filter por. 3 and wash with more diethyl ether (2x100 ml) before drying it in a vacuum oven at 75 °C overnight. After drying, the polymer presented itself as a white powder (1.51g, 78% yield).

2.3.-Hydrogel synthesis

SPLi 6 (0.046g, 0.1 mmol), N,N-DMA (0.195 ml, 1.9 mmol), methylene bis acrylamide (MBA) (0.0063g, 0.04 mmol), and milli-Q water (0.5 ml) are placed in a 4 ml vial and degassed through nitrogen bubbling for 10 minutes. Then, ammonium persulfate (0.0046g, 0.02 mmol) is added to the mixture, which is quickly shaken to ensure full solubilization of the radical initiator and poured into a 100 μm thick custom-made mold. Immediately after, one drop (∼ 50 μL) of TEMED is added to the mixture inside the mold, which is immediately covered with a microscopy glass and kept reacting for 45 minutes. Passed this time, the thin hydrogel is removed from the mold and placed in a beaker containing 200 ml of water to dialyze for 30 minutes. The thin hydrogel is then transferred into a beaker containing 200 ml of 10 mM HCl to dialyze overnight. Passed this time, the hydrogel is dialyzed in water to remove the excess HCl and stored in the dark before using it.

3-UV-Vis absorbance spectroscopy studies

3.1.- UV-Vis of monomer

**General procedure for measurements in methanol**

0.9 mg of SPLi were dissolved in 20 ml of HPLC grade methanol (concentration: 1.9*10^-5^ M). Part of the solution was then treated with DOWEX proton exchange resin to generate a solution of monomer 5 in situ. The solution is then kept in the dark for one hour before measuring. Measurements were performed at 293.15 K (20 °C).

Obtained spectra were analysed using the software Spectragryph, the baseline was corrected for baseline drifting, and then plotted and analysed in Origin Software (from OriginLab).


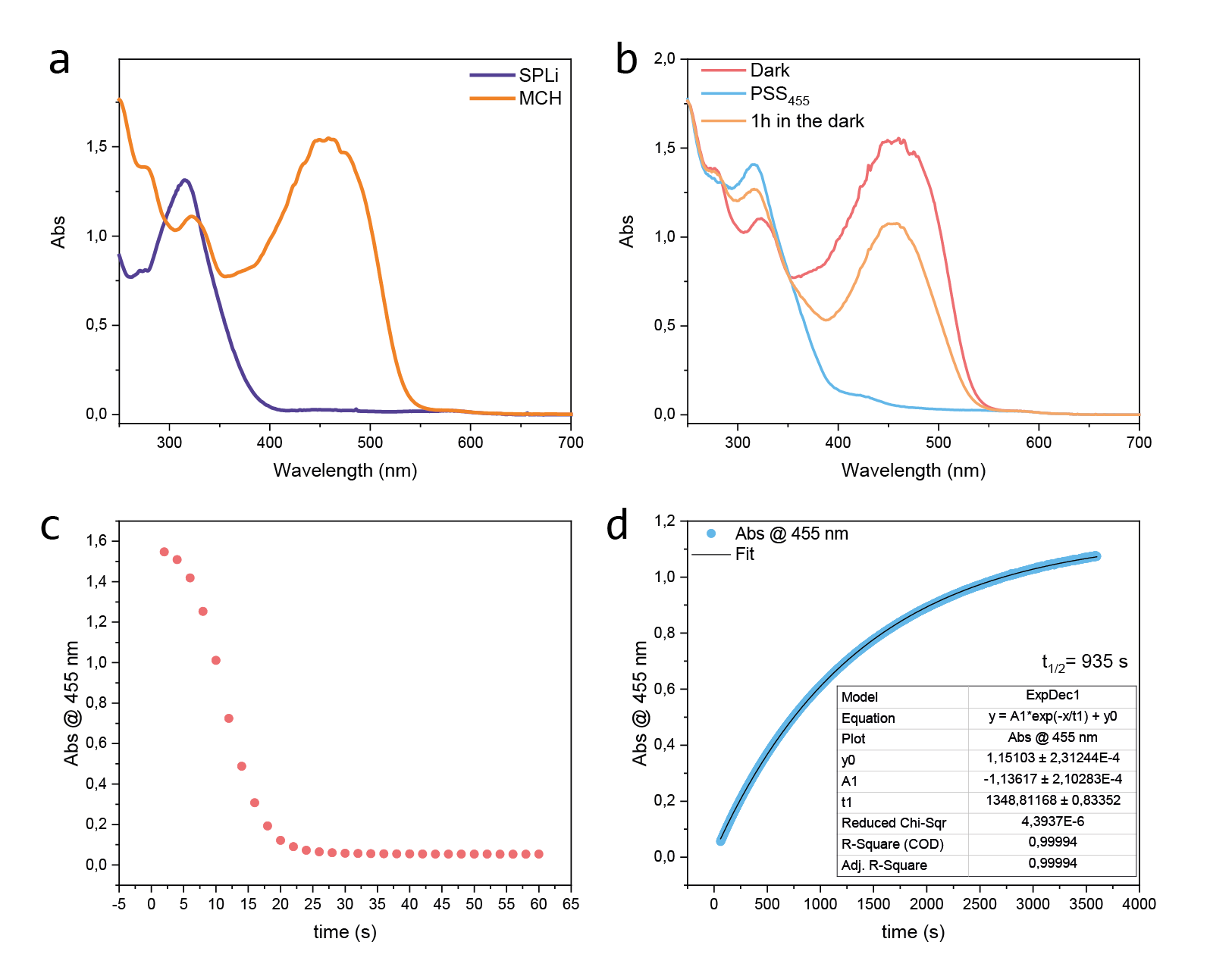


Figure S1: a) Absorbance spectra of 5 (orange) and 6 (purple) in methanol. b) Absorbance spectra of 5 in the dark (red), at PSS455 (cyan), and after storage in the dark for one hour (orange). c) Evolution of the absorbance at 455 nm of 5 upon irradiation with 455 nm light. d) Evolution of the absorbance at 455 nm of 5 upon storage of the irradiated sample in the dark, fitted.

**General procedure for measurements in water**

Samples were prepared from the dilution of a stock solution in Milli-Q water and stored in the dark overnight before measuring. Measurements were performed at 293.15 K (20 °C).

Obtained spectra were analysed using the software Spectragryph, the baseline was corrected for baseline drifting and then plotted and analysed in Origin Software (from OriginLab).


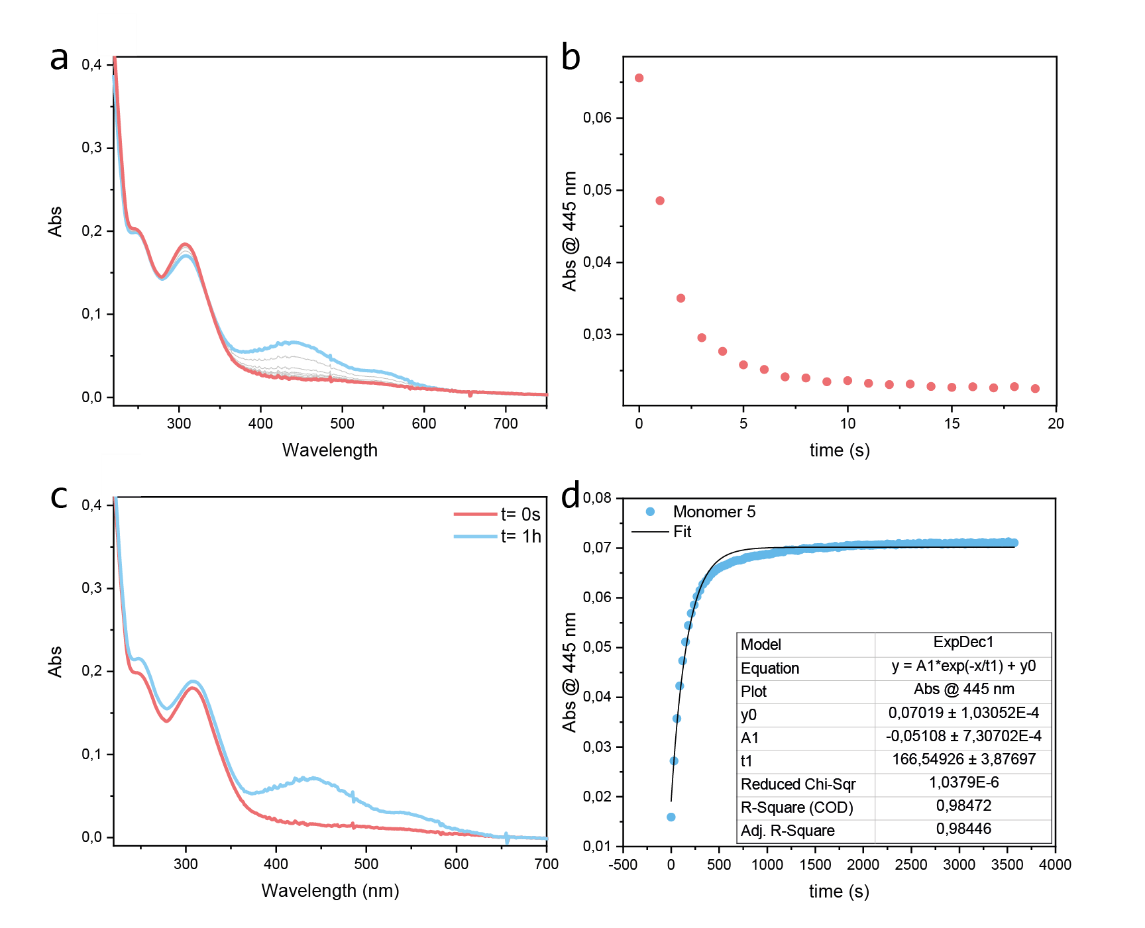
Figure S2: a) Evolution of the UV-Vis absorbance spectra of merocyanine metastable-state photoacid 5 upon irradiation with 455 nm LED (cyan= dark, red= PSS455). Characteristic absorbance peaks for MCH (centred at 445 nm) and for its deprotonated isomer MC (centred at 550 nm) can be spotted. b) Evolution of the absorbance at 445 nm during irradiation with a 455 nm LED of monomer 5 in water. c) Evolution of the UV-Vis absorbance spectra of merocyanine metastable-state photoacid 5 after irradiation with 455 nm LED upon storage in the dark. d) Evolution of the absorbance at 445 nm upon storage in the dark of a previously irradiated solution of monomer 5.

3.2.- UV-Vis of polymers in solution

**General procedure for measurements in water of polymers**

Samples were prepared by dissolution of p-SPLi-n polymers in milli-Q water (see Table S1). 3 ml of the solutions were then treated with DOWEX proton exchange resin to generate p-MCH-n in solution and stored in the dark overnight before measuring. Measurements were performed at 293.15 K (20 °C).

Obtained spectra were analysed using the software Spectragryph, the baseline was corrected for baseline drifting and then plotted and analysed in Origin Software (from OriginLab). The obtained absorbance spectra were normalized at the isosbestic point (317 nm).

Table S2: quantities for the preparation of solutions of polymers for UV-Vis absorbance spectroscopy.

|  | mass (g) | volume (ml) |
| --- | --- | --- |
| p-SPLi-1 | 0.0032 | 10 |
| p-SPLi-2 | 0.0062 | 10 |
| p-SPLi-3 | 0.0124 | 10 |
| p-SPLi-4 | 0.0245 | 10 |


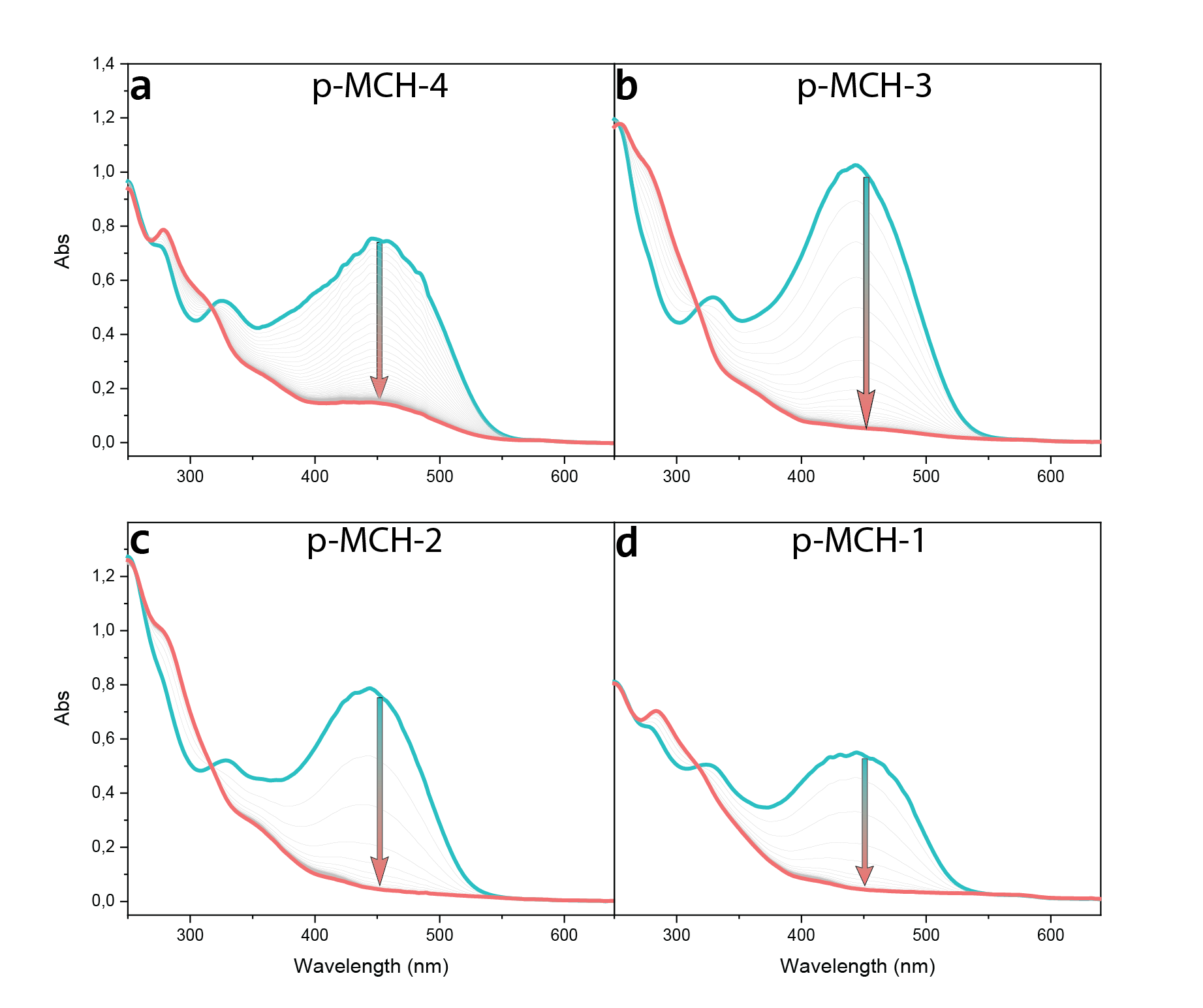


Figure S3: Evolution of the UV-Vis absorbance spectra of a) p-MCH-4, b) p-MCH-3, c) p-MCH-2, d) p-MCH-1 upon irradiation with blue light in water (cyan= dark, salmon= PSS455). Spectra are normalized at the isosbestic point (317 nm).


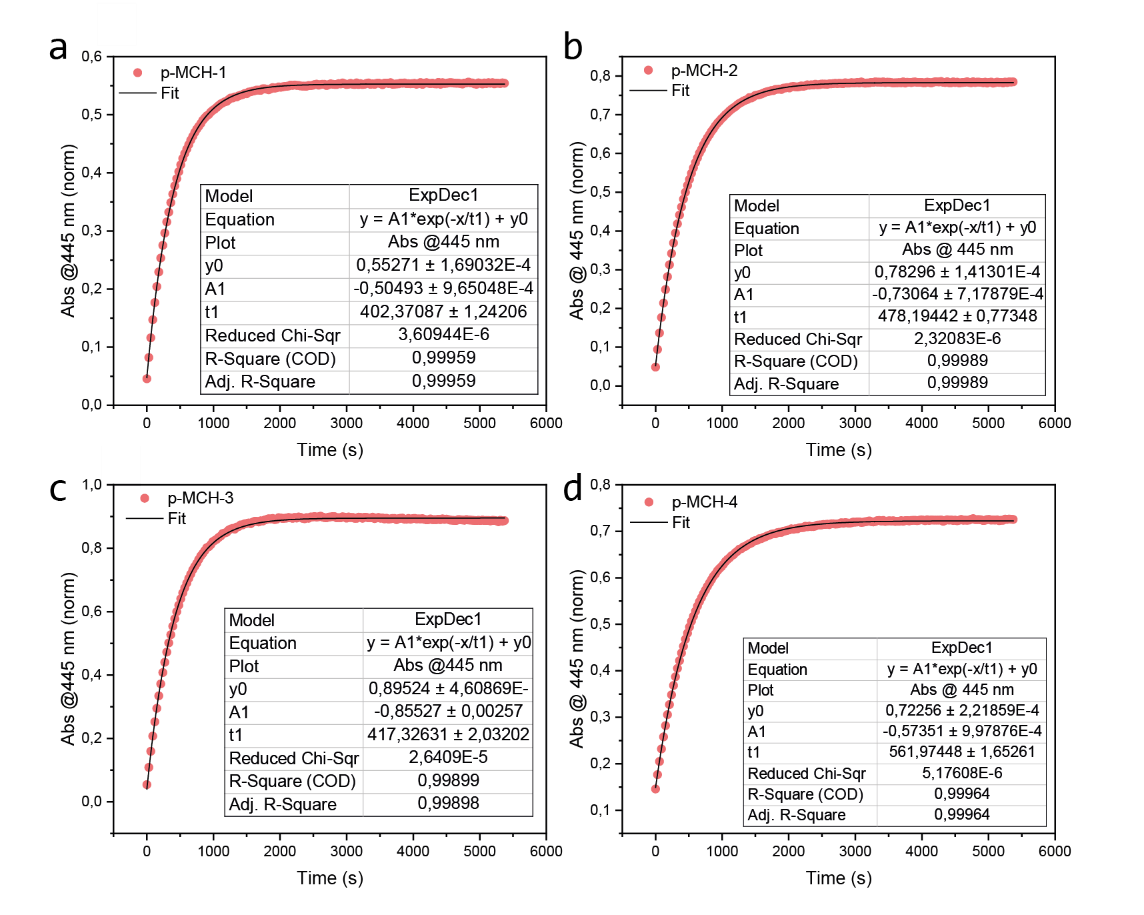

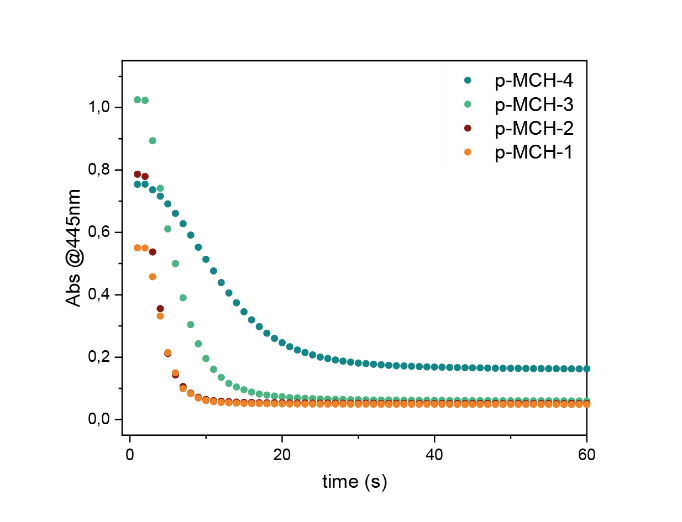
Figure S4: change in the absorbance at 445 nm upon irradiation with a 455 nm LED of p-MCH-n in water.

Figure S5: evolution of the absorbance of a) p-MCH-1, b) p-MCH-2, c) p-MCH-3, d) p-MCH-4 after irradiation with 455 nm LED, upon storage in the dark.

3.3.- UV-Vis of polymers in the solid state

**General procedure**

30 μL of a solution of p-MCH-n in methanol (10 mg/ml) are drop cast on the inside wall of a quartz cuvette placed on a hot plate at 60 °C. The film is left drying for 5 minutes, and then a small flat piece of cotton wool is placed at the bottom of the cuvette in a way that it doesn’t interfere with the UV-Vis absorbance measurements. The sample is stored in the dark overnight before measuring. Before measuring the sample, to ensure high humidity conditions, 100 μL of MilliQ water are placed inside the cuvette, taking care to let it be absorbed by the cotton wool at the bottom. After that, the cuvette is sealed and allowed to equilibrate for 30 minutes.

Irradiation experiments are performed by using a custom-made 3D-printed holder capable of keeping an airtight environment inside the cuvette, and at the same time to allow irradiation from the top of the cuvette, directly onto the drop-casted polymeric film.

The obtained absorbance spectra were normalized at the isosbestic point (319 nm).


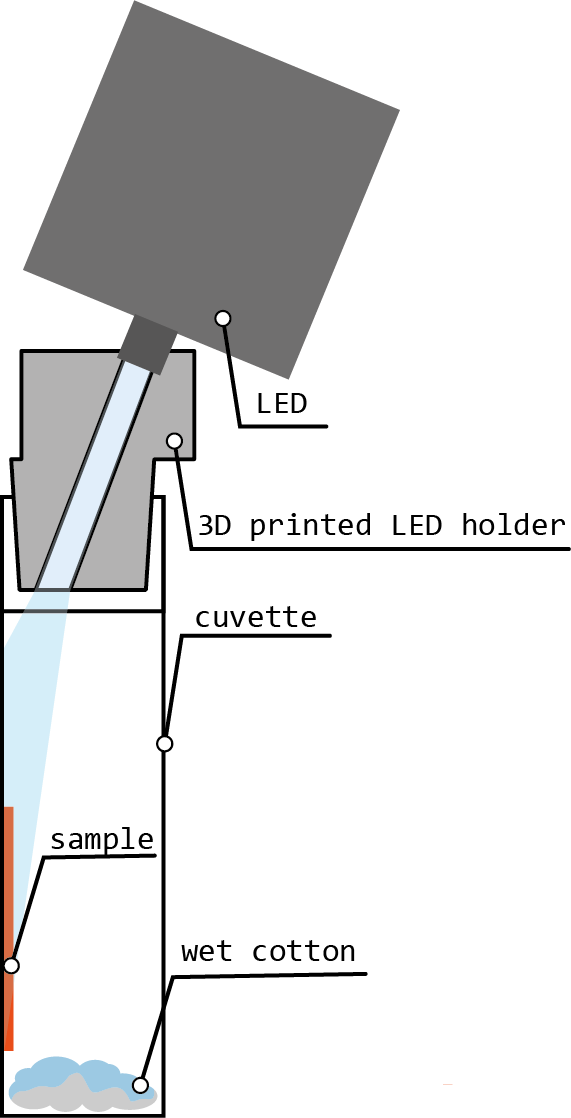


Scheme S2: representation of the setup used for solid-state UV-Vis absorbance measurements on polymeric films.


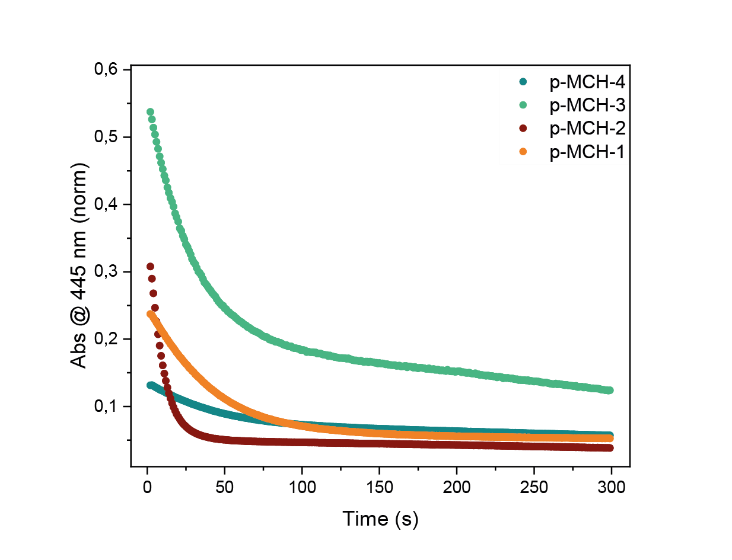
Figure S6: change in the absorbance at 445 nm upon irradiation with a 455 nm LED of p-MCH-n in the solid state.

4- Polymers characterization

4.1.- Thermo Gravimetric Analysis (TGA)

Thermogravimetric analysis (TGA) was performed on a Perkin Elmer STA 6000 instrument under a continuous nitrogen flow (30 ml/min). Samples have been heated from 30 to 700 °C at a heating rate of 10 °C/min.


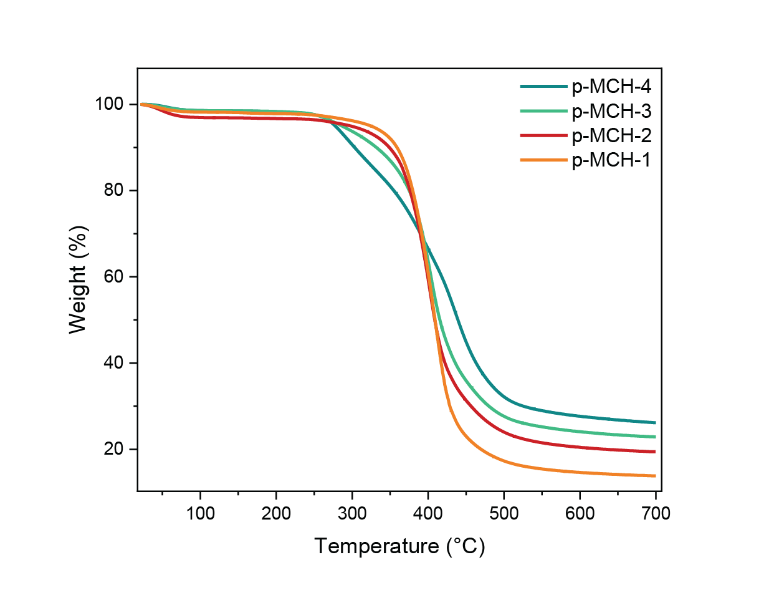


Figure S7: TGA traces of p-MCH-n.

4.2.- Gel Permeation chromatography (GPC)

**Sample preparation**

p-SPLi-n is dissolved in 1 ml of DMF+LiBr to a concentration varying from 2 to 5 mg/ml and treated with DOWEX proton exchange membrane. The sample is then filtered using a syringe filter with a 200 μm size and kept in the dark before the measurement.

Table S3: Mn and PDI of the prepared p-MCH-n polymers.

|  | Mn | PDI |
| --- | --- | --- |
| p-MCH-1 | 12033 | 2.60 |
| p-MCH-2 | 8768 | 3.76 |
| p-MCH-3 | 18606 | 2.04 |
| p-MCH-4 | 16828 | 1.53 |
| p-N,N-DMA | 16013 | 3.70 |
|  |  |  |


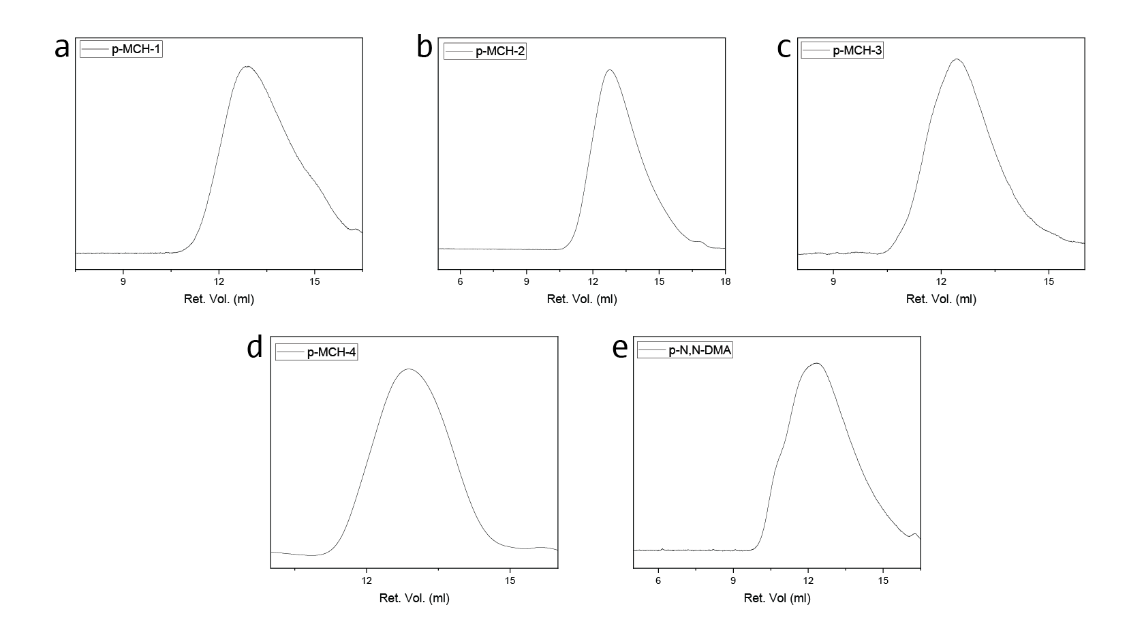
Figure S8: GPC chromatograms of a) p-MCH-1, b) p-MCH-2, c) p-MCH-3, d) p-MCH-4, e) p-N,N-DMA.

5- Electrochemical Impedance Spectroscopy

5.1.- EIS with in-situ irradiation

Solutions preparation: to a 10 mg/ml solution of p-SPLi-n in methanol, DOWEX proton exchange resin is added. The sample is vigorously shaken using a vortex for one minute, after which the solution is filtered through a 200 μm syringe filter, yielding the desired solution of p-MCH-n.

**Film preparation**

Films of p-MCH-n were prepared by casting 80 μL of solution on IDE electrodes at 55 °C. The films have been left drying on the hot plate for one or two minutes, until the methanol has completely evaporated, then placed in a controlled humidity chamber in the dark at RH 100% and 20 °C to equilibrate overnight before measurements.

**Setup description**

The sample is placed into the controlled humidity chamber at RH= 100 overnight in the dark to equilibrate. Wet cotton wool is placed inside the sealed chamber in a way that it doesn’t directly touch the sample, to achieve RH=100. The LED is placed at 9 cm from the glass window of the controlled humidity chamber. The LED power is set at 0.5 A. An air nozzle is placed in a way that room-temperature air is blown directly on the glass window of the controlled humidity chamber, to avoid heating during the irradiation.

**Irradiation experiments**

The first EIS scan was performed in the dark, and then the LED and airflow were turned on and kept on for the duration of the experiment. After, the LED was turned off and the window on the cell was covered again to allow the sample to relax in the dark, while additional EIS scans were performed, to follow the change in resistance during the back relaxation process. Impedance experiment in the dark and under illumination have been repeated three times on three different films per each p-MCH-n polymer to ensure reproducibility of the results and evaluate the error on the characteristic conductance of each polymer.

Figure S9: switching capability of p-MCH-n series obtained from the equation: $\frac{(\sigma dark-\sigma irrad)}{\sigma irrad}$


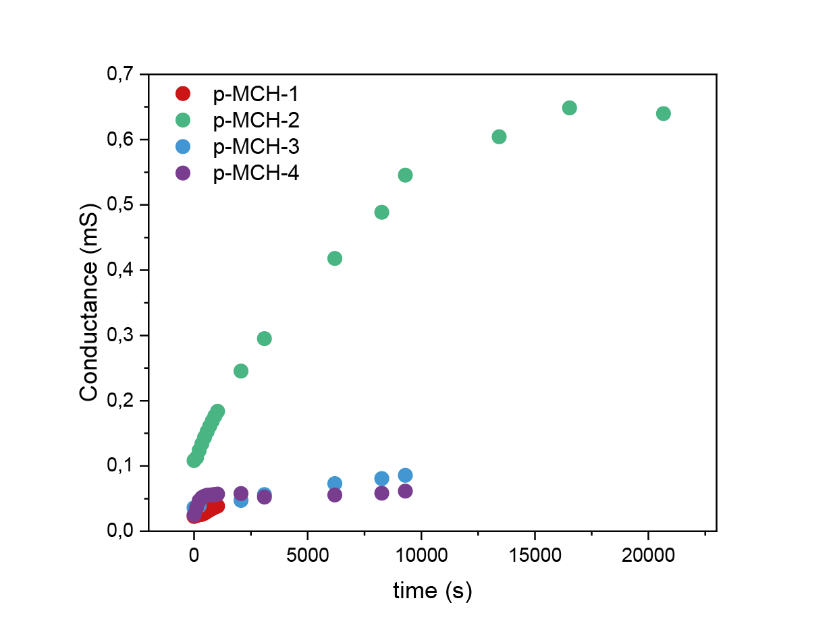


Figure S10: evolution of the conductance of p-MCH-n upon storage in the dark after their irradiation.

5.2.- Arrhenius analysis

Arrhenius analysis has been performed not on the polymeric thin film samples, but on the MCH hydrogel instead. This is because the thin films are extremely subjected to dehydration upon heating, and for this reason, their conductivity is influenced by the loss of water, rather than the increase in temperature.

**General procedure**

The MCH hydrogel has been secured to the IDE electrode using Kapton tape and then immersed in water. The sample has been kept in the dark for the duration of the experiment, and the temperature of the water bath has been increased stepwise to acquire the different data for the analysis.

From the Arrhenius equation σ= σ_0​_ x e^-(Ea/RT)^ we can derive the activation energy (E_a_).

E_a_= -(slope x R) = 15.63 kJ/mol = 0.162 eV

(R= 8.3148 J/mol x K)

The calculated value is in good agreement with a Grotthus-like mechanism (E_a_≈0.1−0.4 eV).


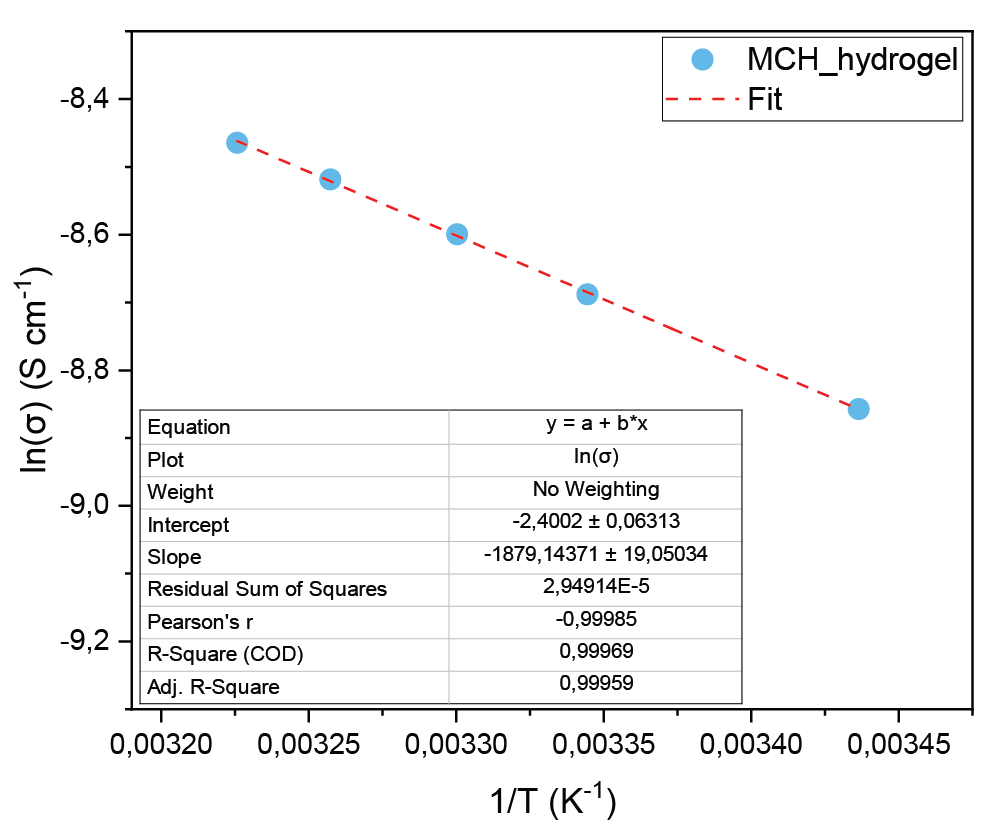


Figure S11: Arrhenius plot of MCH hydrogel in the dark, fitted.

6-Irradiation experiment on MCH hydrogel


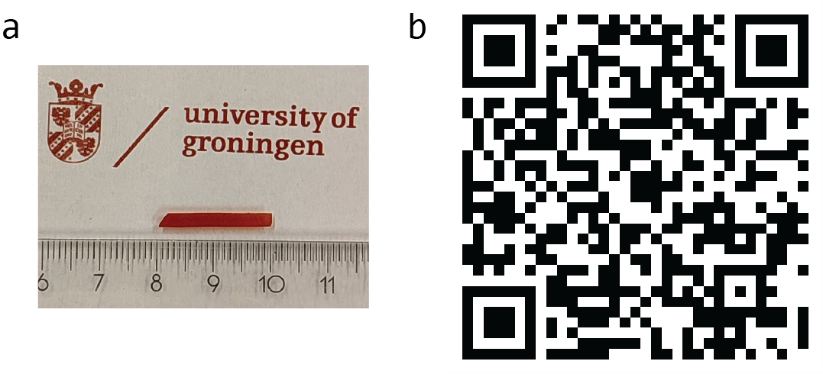
The actuation experiment of MCH hydrogel has been conducted as follows: the hydrogel strip (Figure S11a) has been secured to a pair of tweezers and immersed in Milli-Q water inside a quartz cuvette, having care that the flat side of the gel is facing one of the four walls of the cuvette. The sample is left in the dark for one hour to equilibrate. Passed this time, 455 nm light is shined onto the face of the hydrogel, and the experiment is recorded (Supplementary Video 1).

Figure S12: Picture of the hydrogel strip used for actuation experiment.

7- Grazing incident wide angle x-ray scattering (GIWAXS)

**Sample preparation**

Samples preparation: 0,06 ml of a 10 mg/ml solution of p-MCH-n are casted on a silicon wafer at 60 °C and stored in the dark. The measurements have been performed in a vacuum chamber.

8- Quantum mechanical calculations

All quantum chemical calculations were performed using the Q-Chem 6.2 software.^[2]^

| 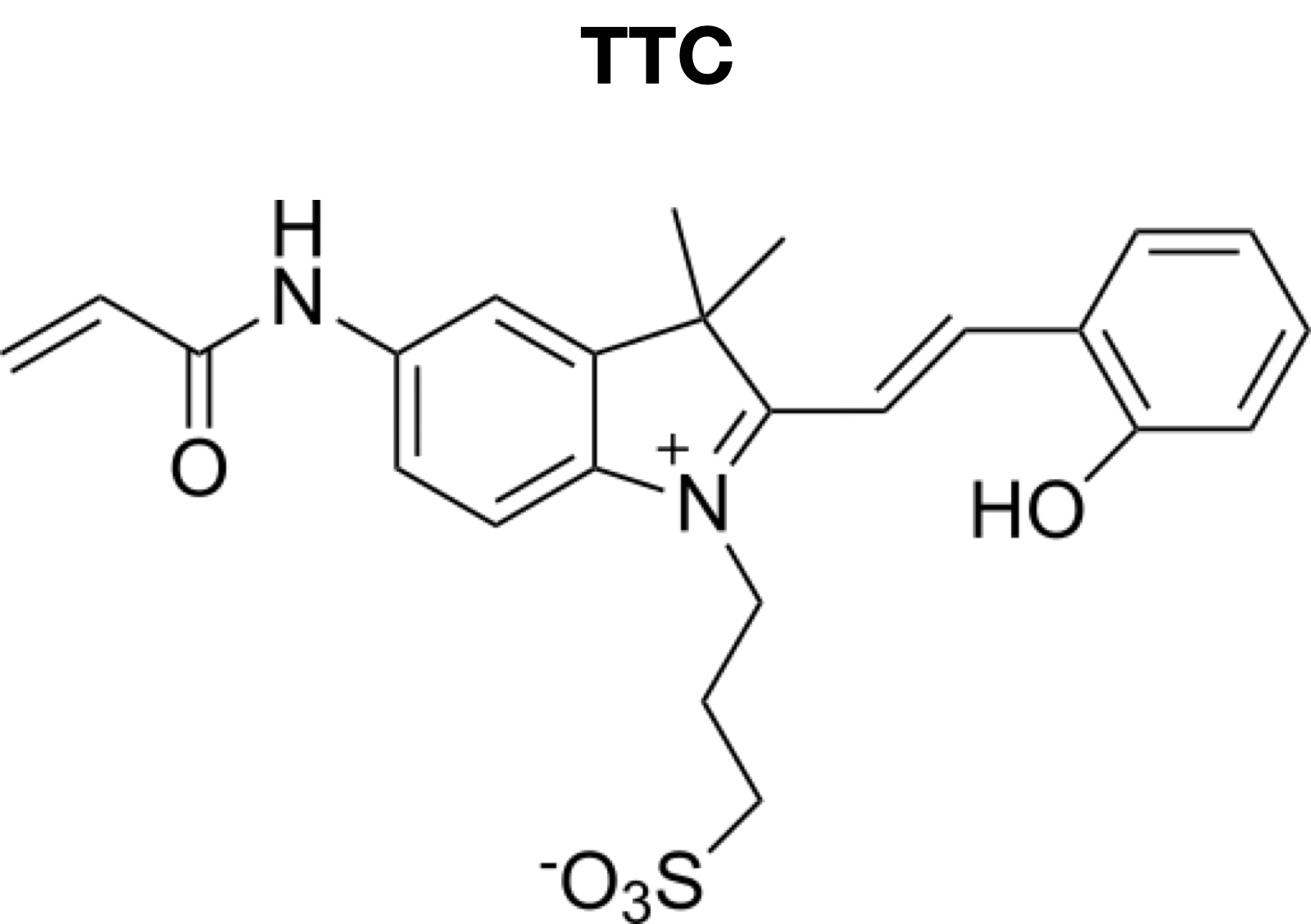 | 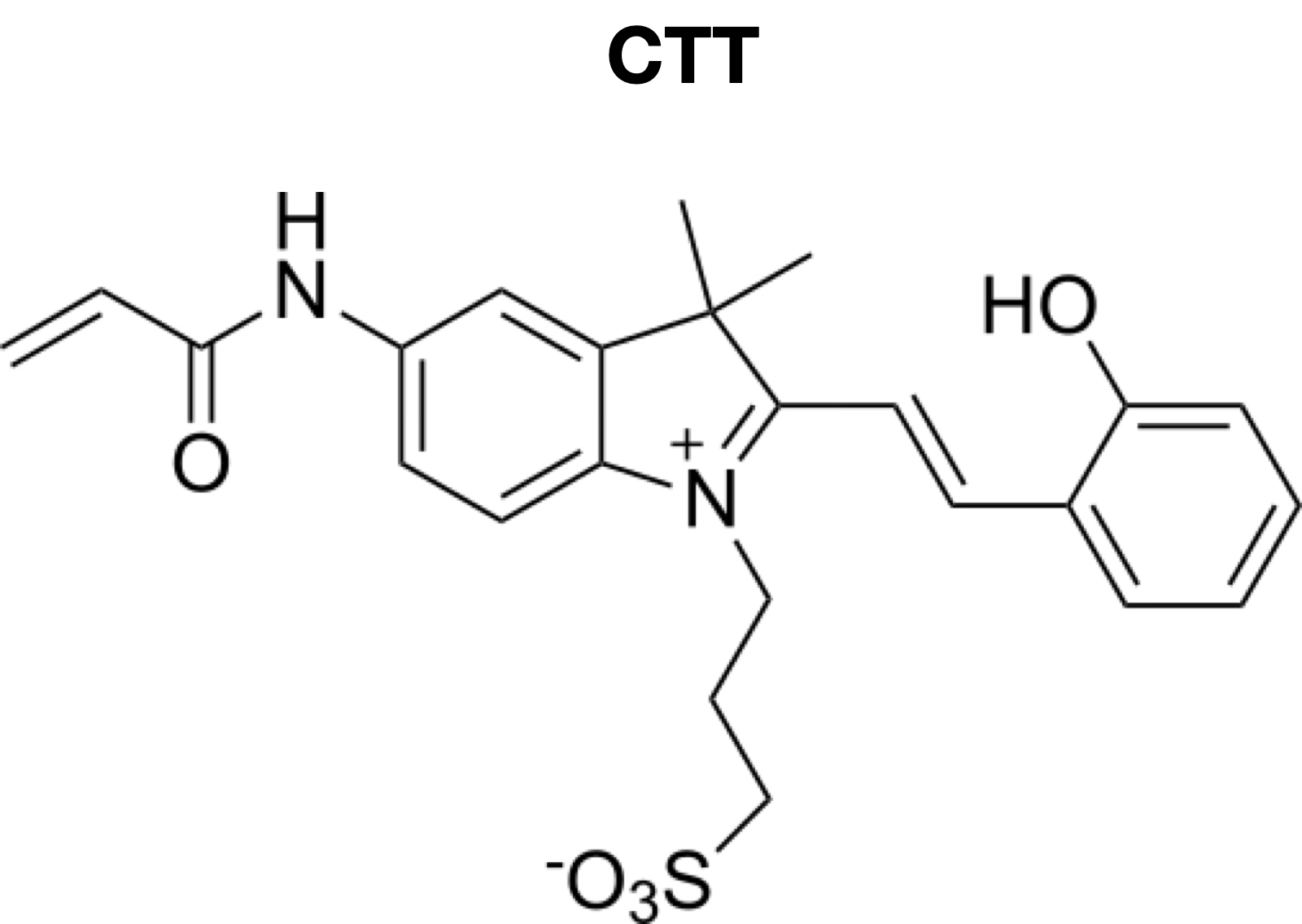 |
| --- | --- |
| 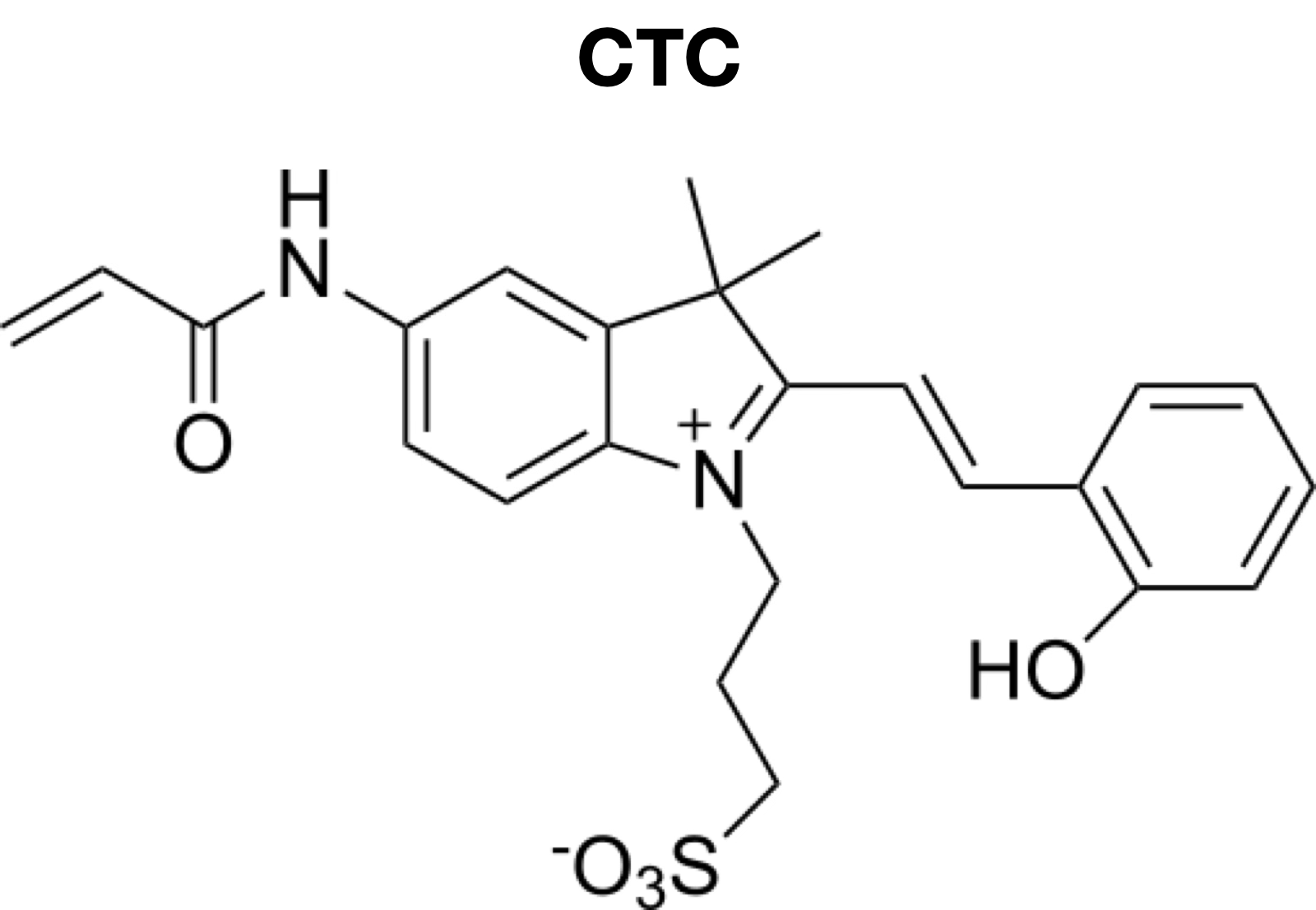 | 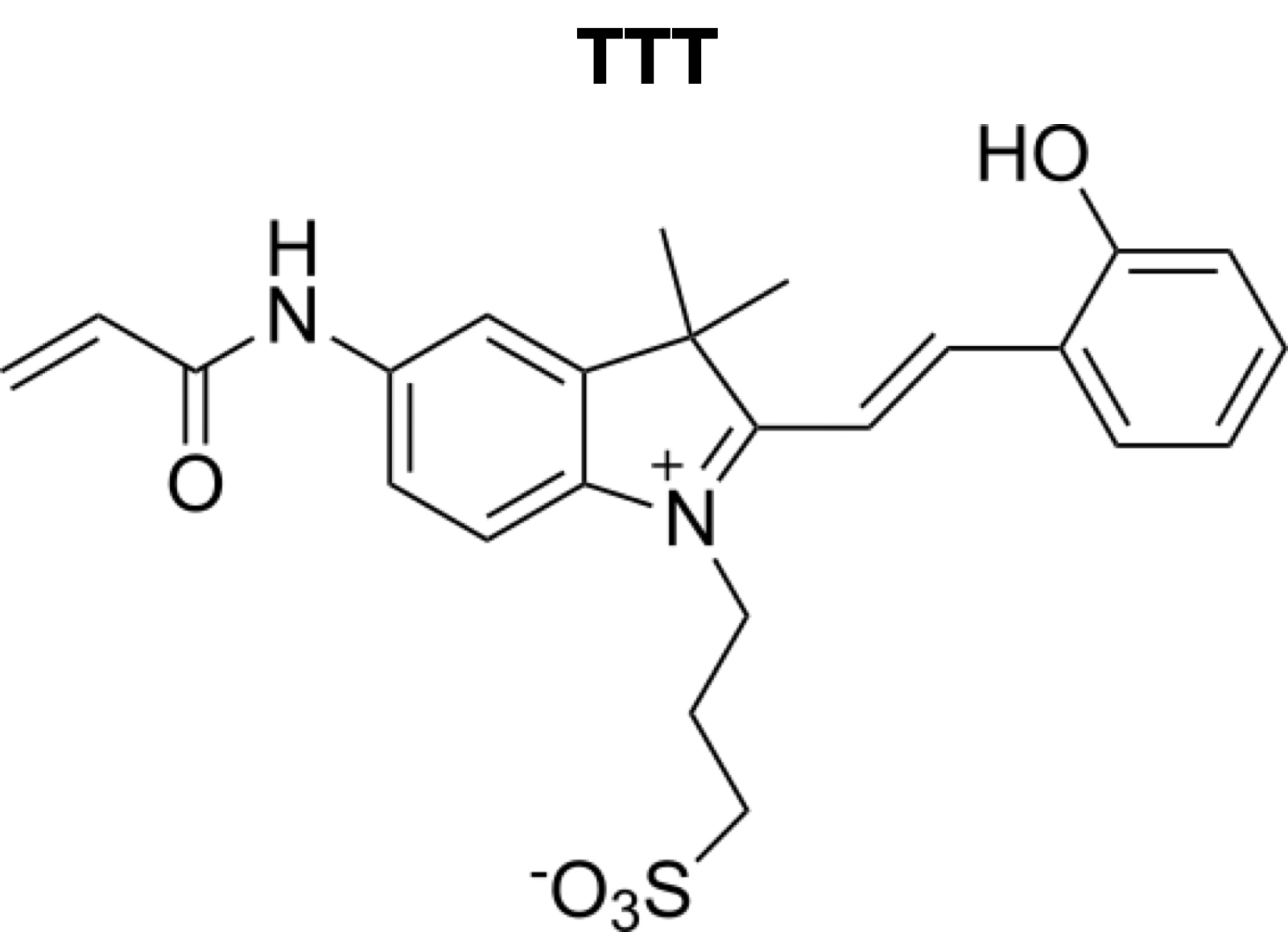 |

Figure S13: Possible conformations of the MCH molecule, representing different cis-(C)/trans-(T) conformers, referring to the configurations of the central methine bridge.


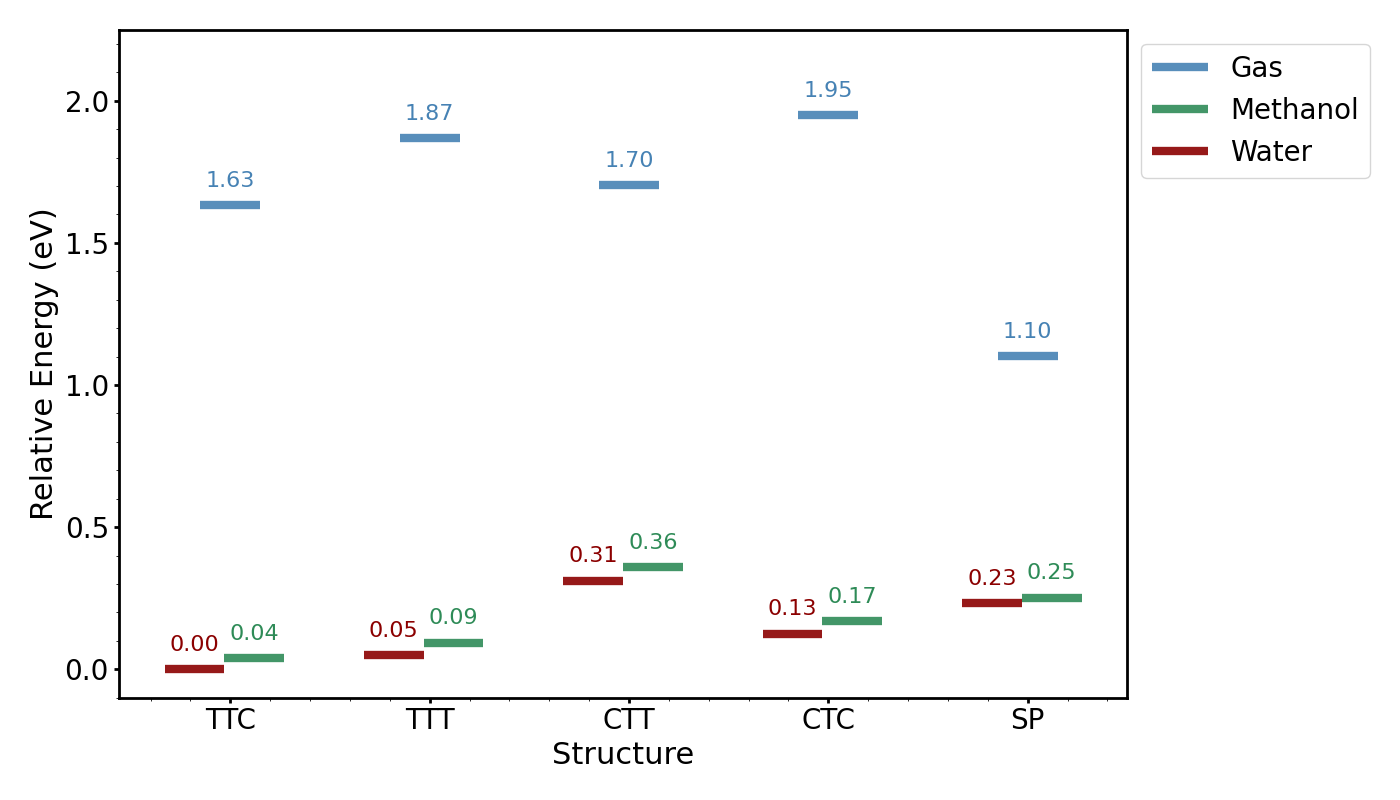


Figure S 14: Energies (in eV) relative to the ground-state energy of the open-ring TTC structure in water calculated at the ωB97X-D/cc-PVDZ.


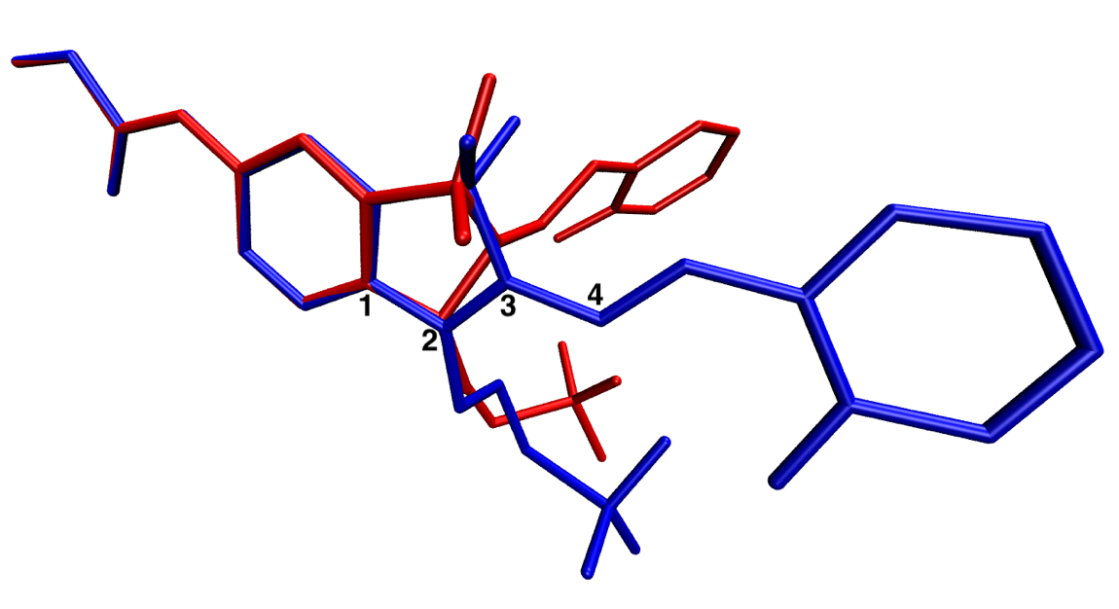
Figure S15. Comparison of MCH (blue) and MECP S2/S2 (red) geometries illustrating the definition of the dihedral angle φ (1–2–3–4).


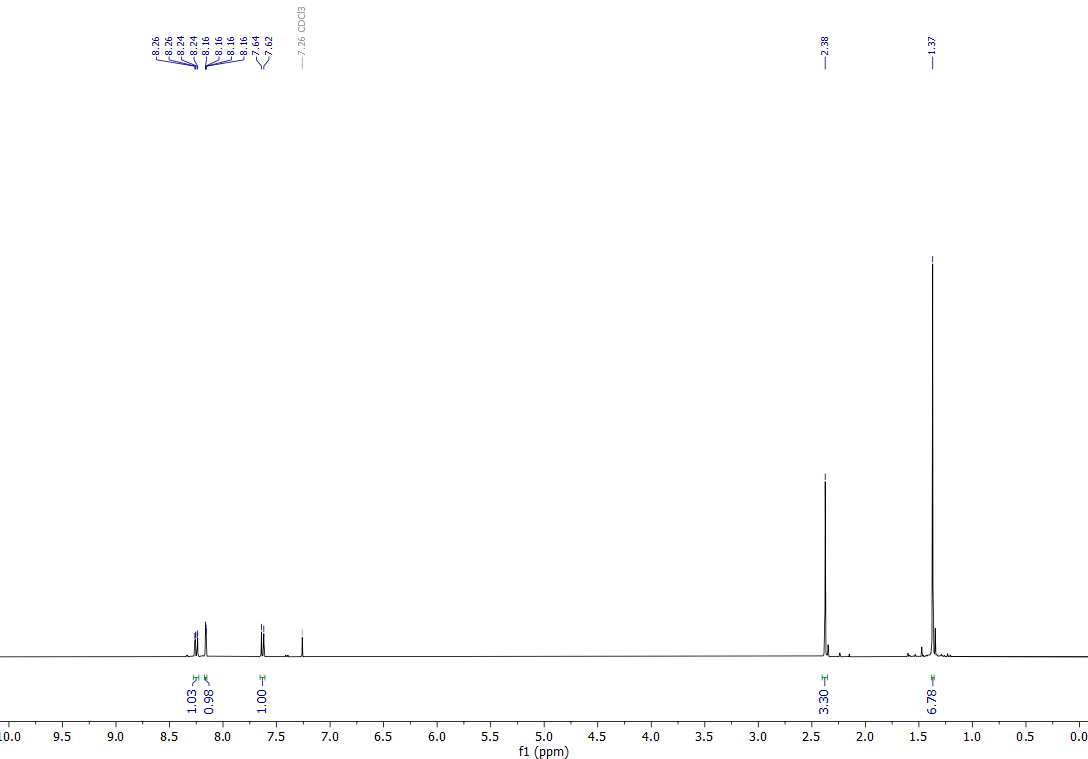
9- NMR spectra


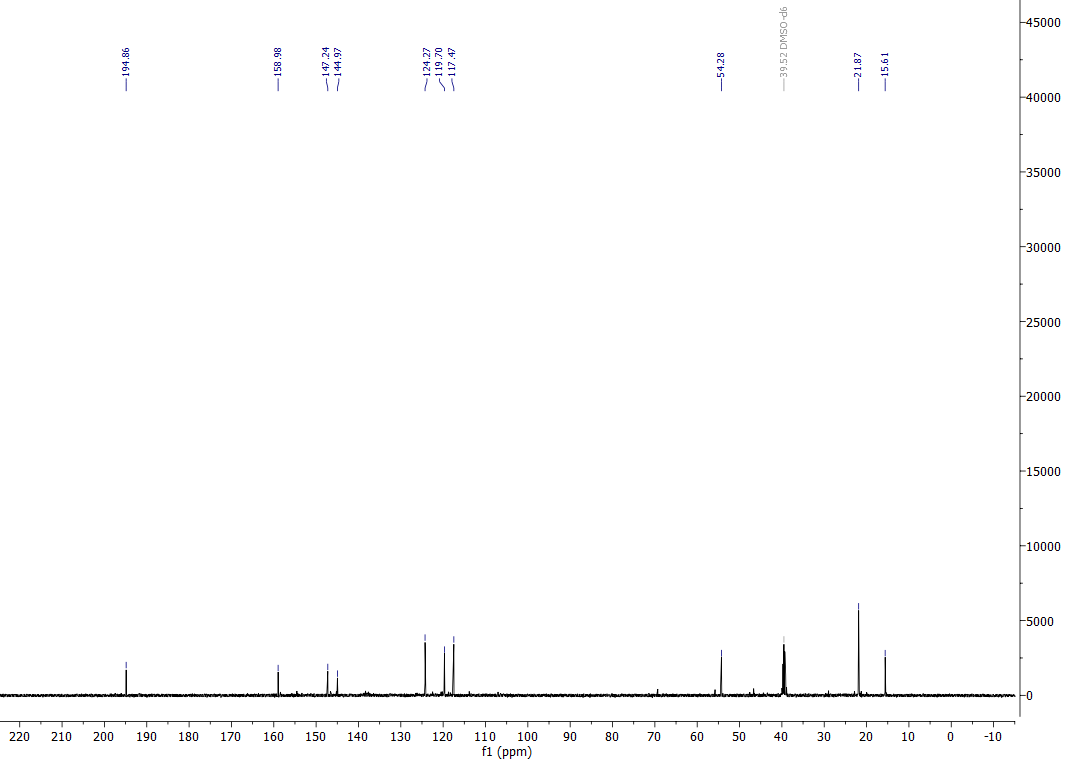
Figure S16: ^1^H-NMR spectra of 1 in CDCl_3_

Figure S17: ^13^C-NMR spectra of 1 in CDCl_3_


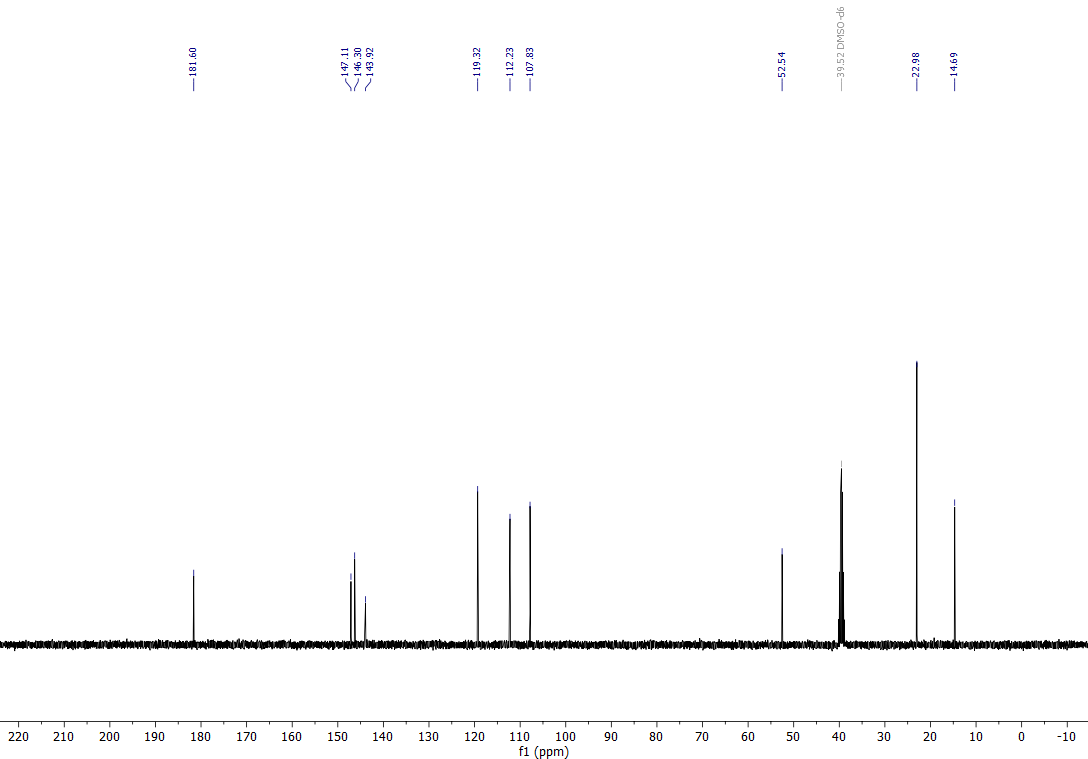

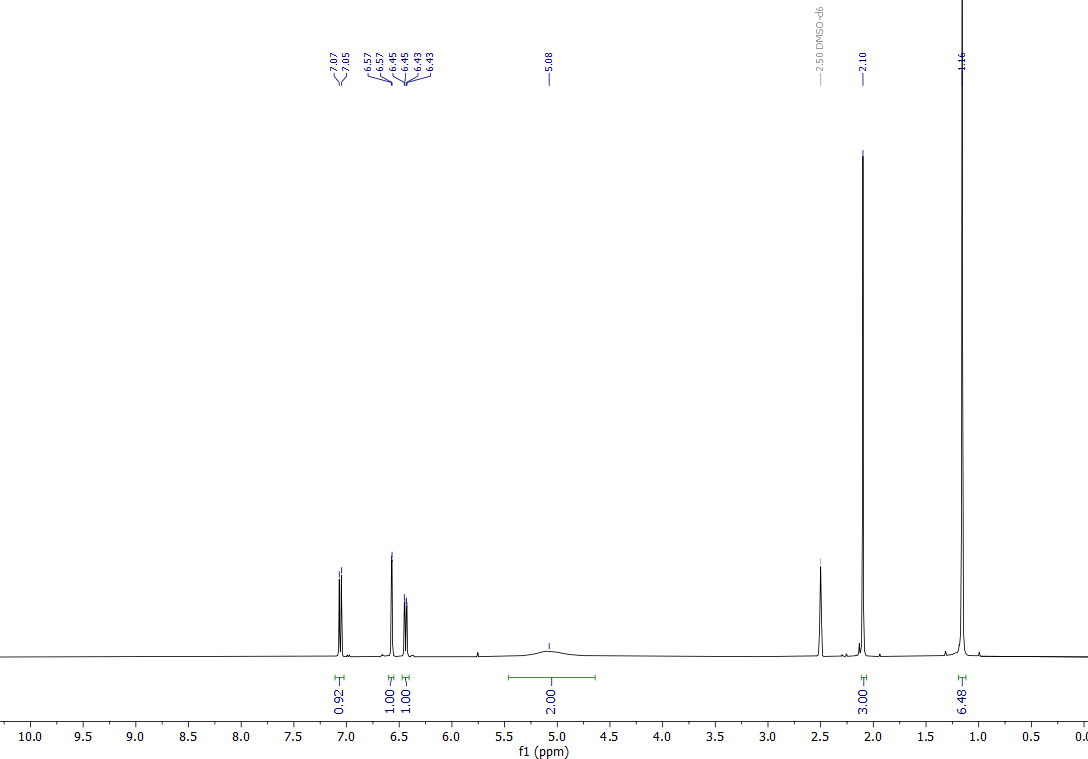
Figure S18: ^1^H-NMR spectra of 2 in DMSO-d6

Figure S19: ^13^C-NMR spectra of 2 in DMSO-d6


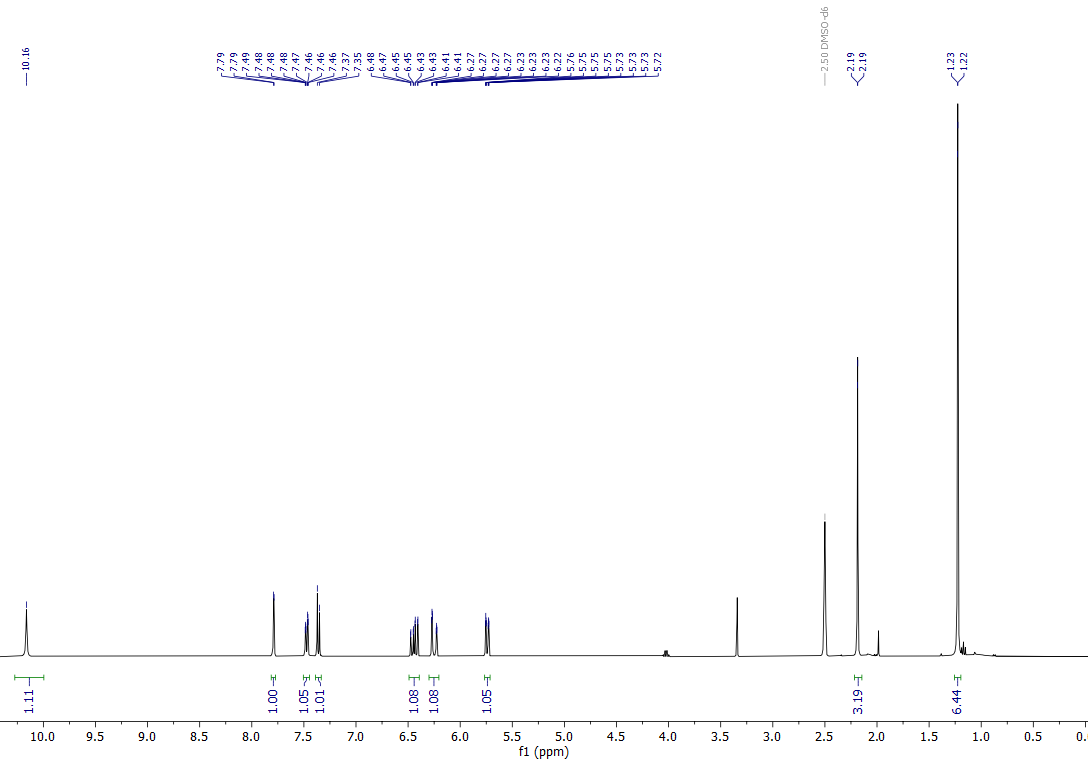
Figure S20: ^1^H-NMR spectra of 3 in DMSO-d6


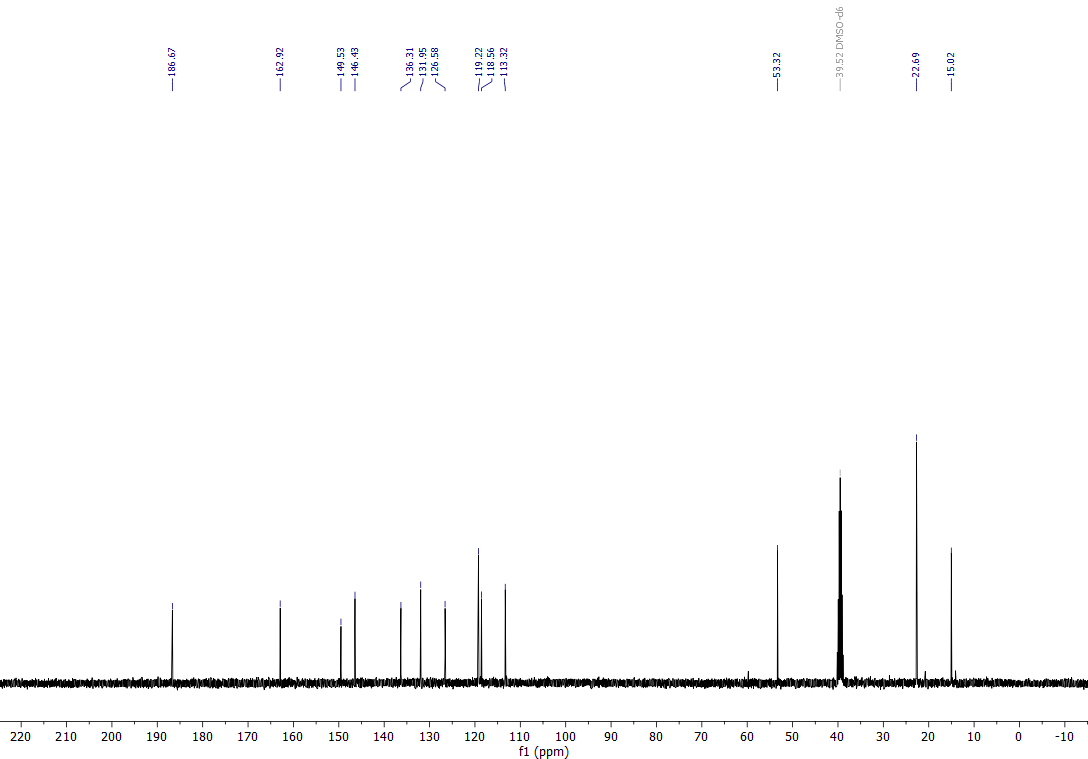


Figure S21: ^13^C-NMR spectra of 3 in DMSO-d6


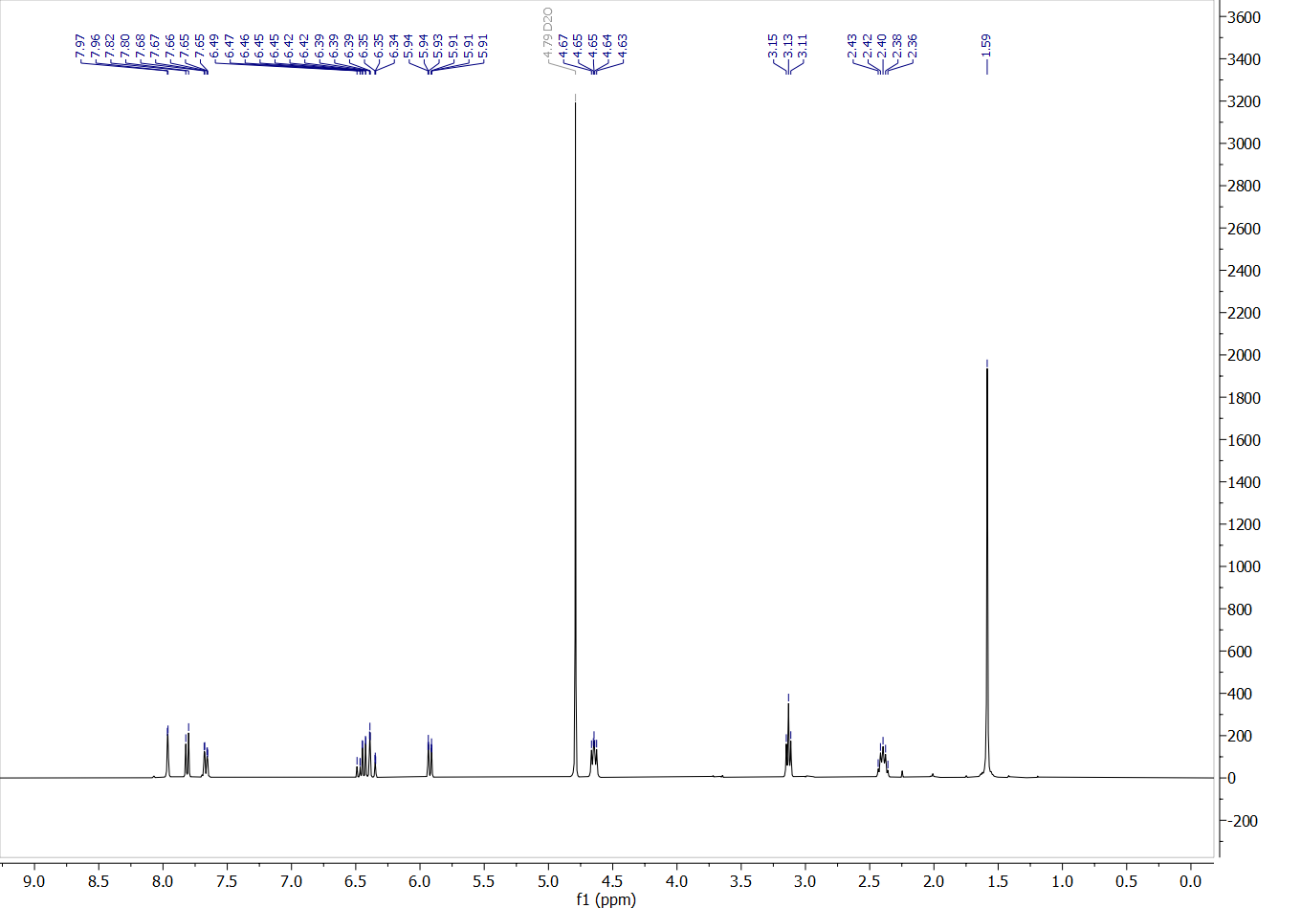
Figure S22: ^1^H-NMR spectra of 4 in D_2_O + LiCl


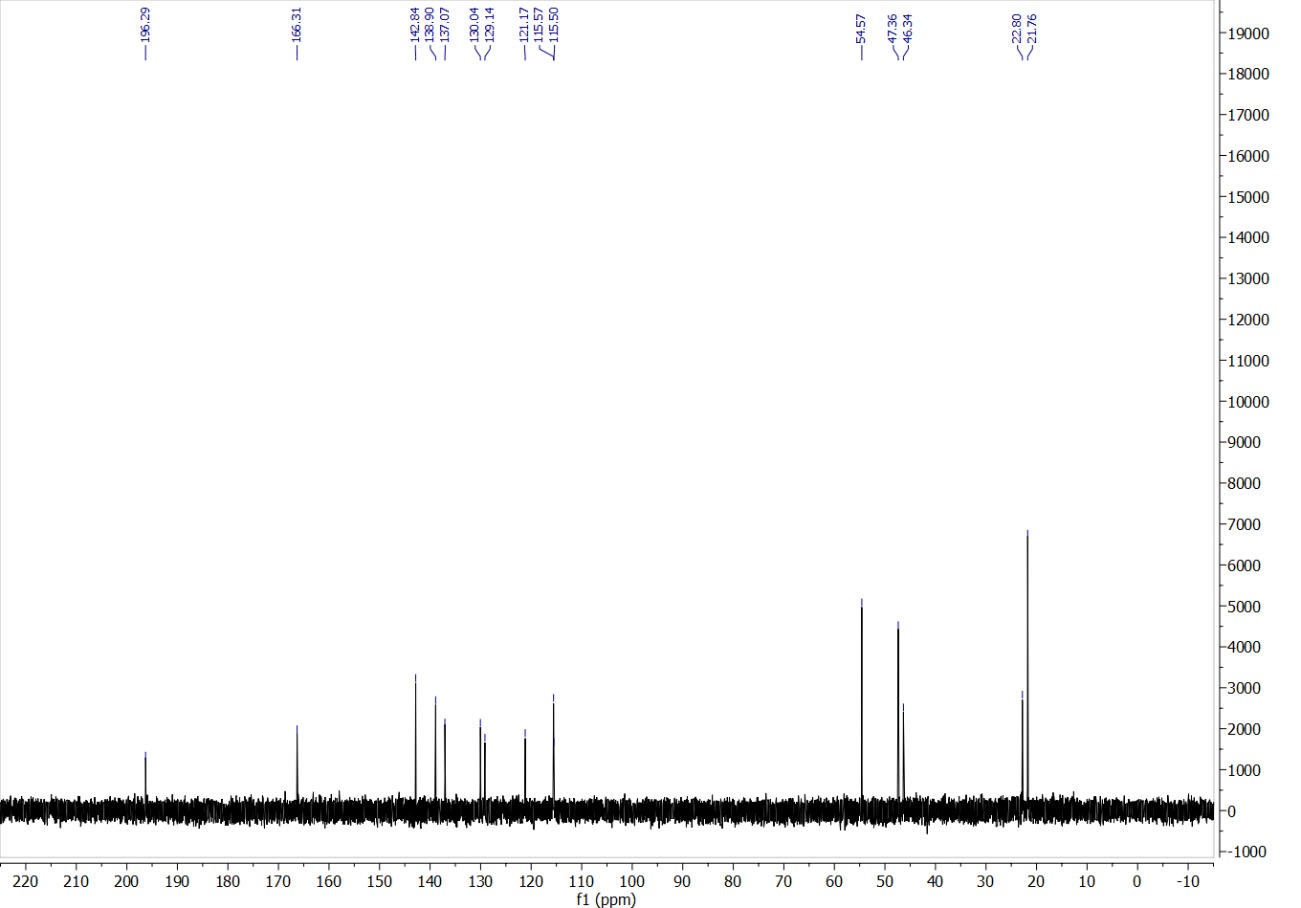


Figure S23: ^13^C-NMR spectra of 4 in D_2_O + LiCl


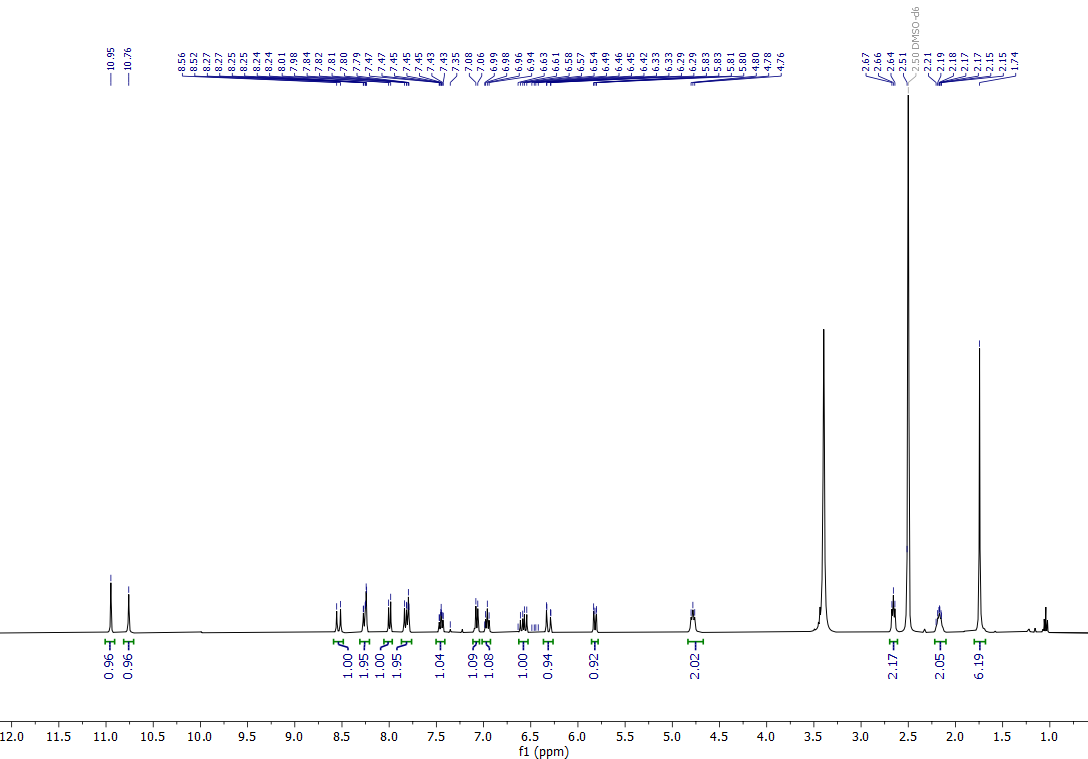
Figure S24: ^1^H-NMR spectra of 5 in DMSO-d6


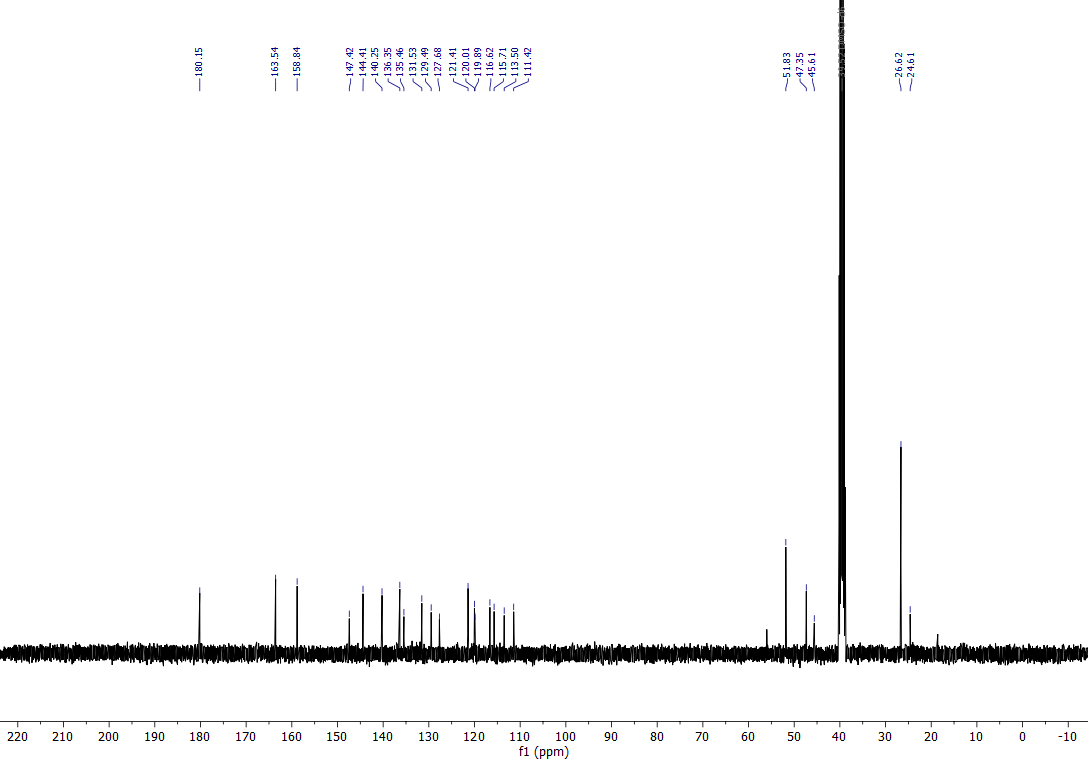


Figure S25: ^13^C-NMR spectra of 5 in DMSO-d6


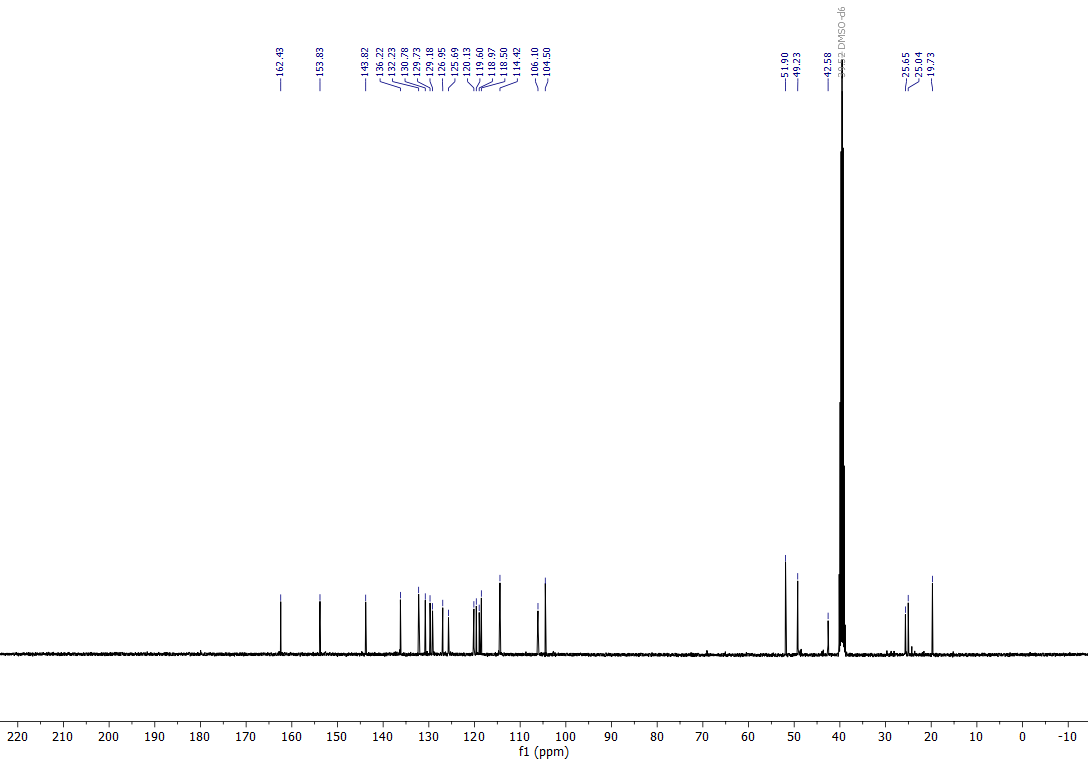

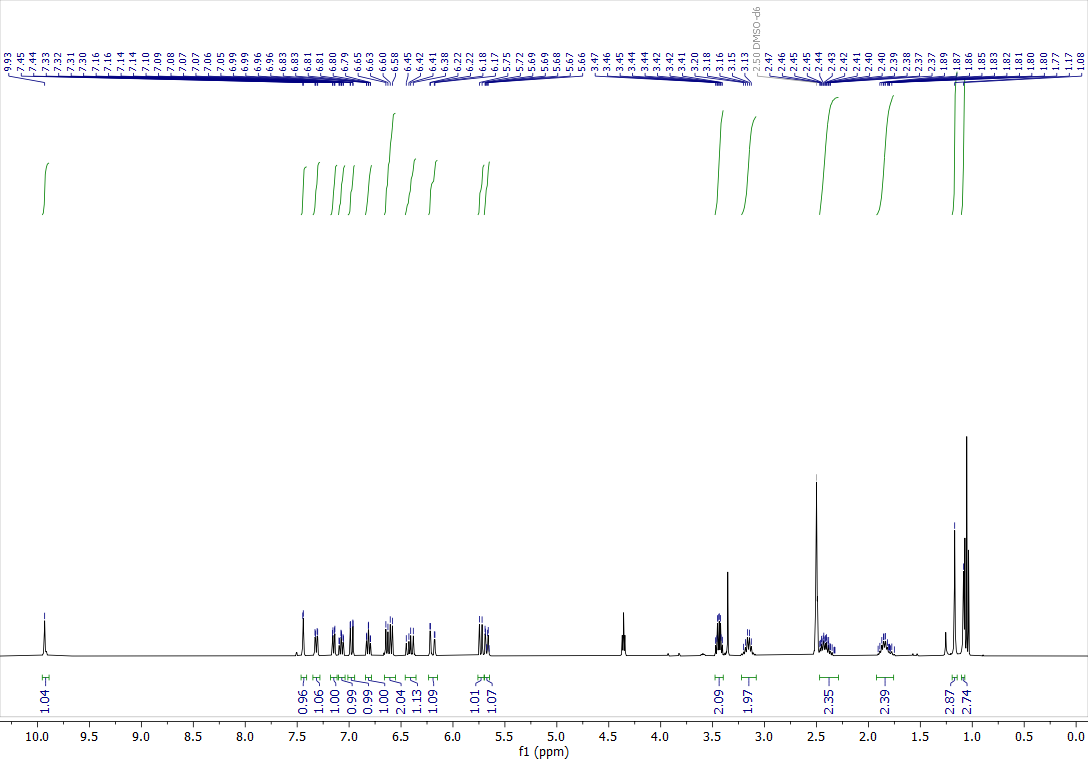
Figure S26: ^1^H-NMR spectra of 6 in DMSO-d6

Figure S27: ^13^C-NMR spectra of 6 in DMSO-d6


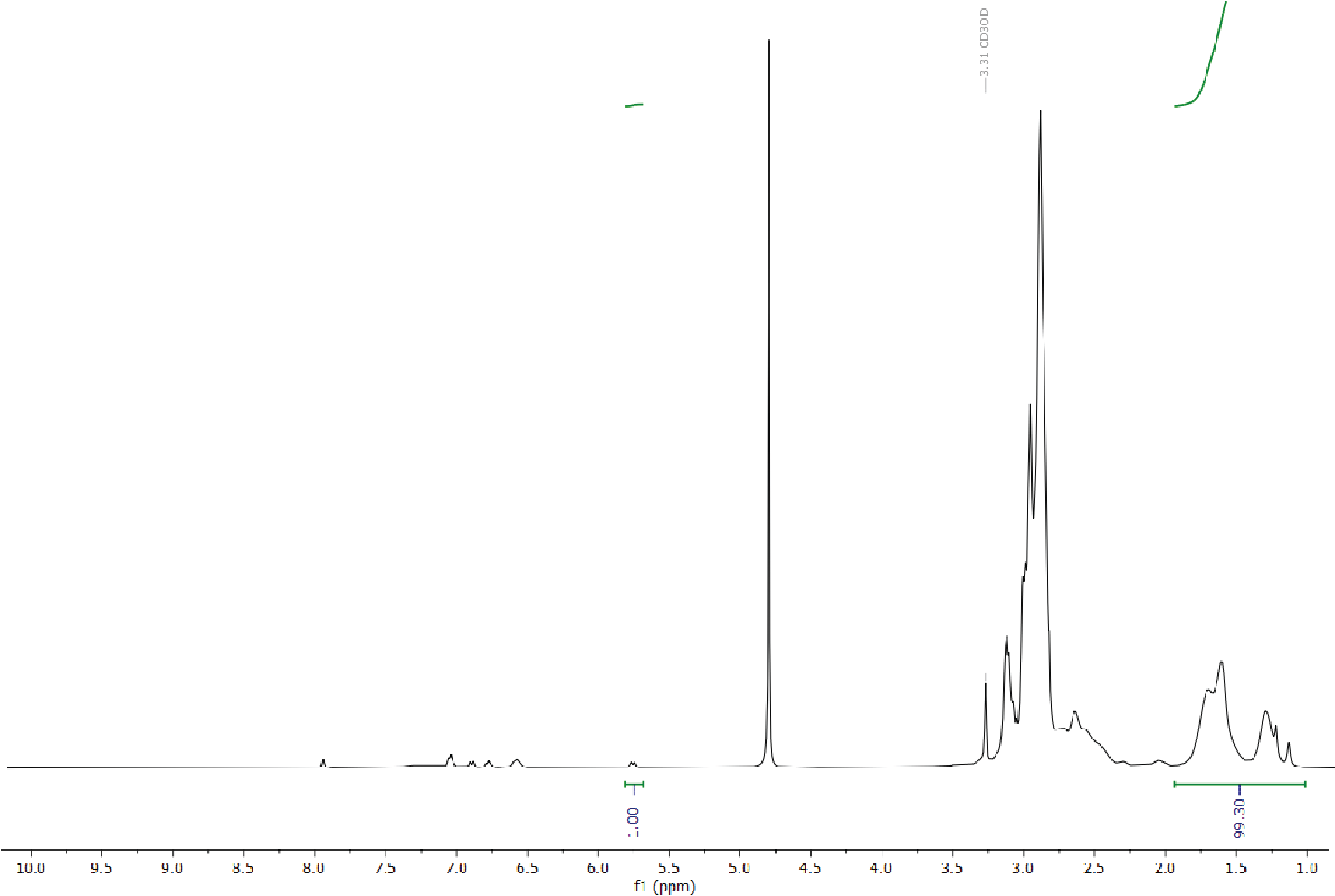
Figure S28: ^1^H-NMR spectra of p-SPLi-1 in CD_3_OD


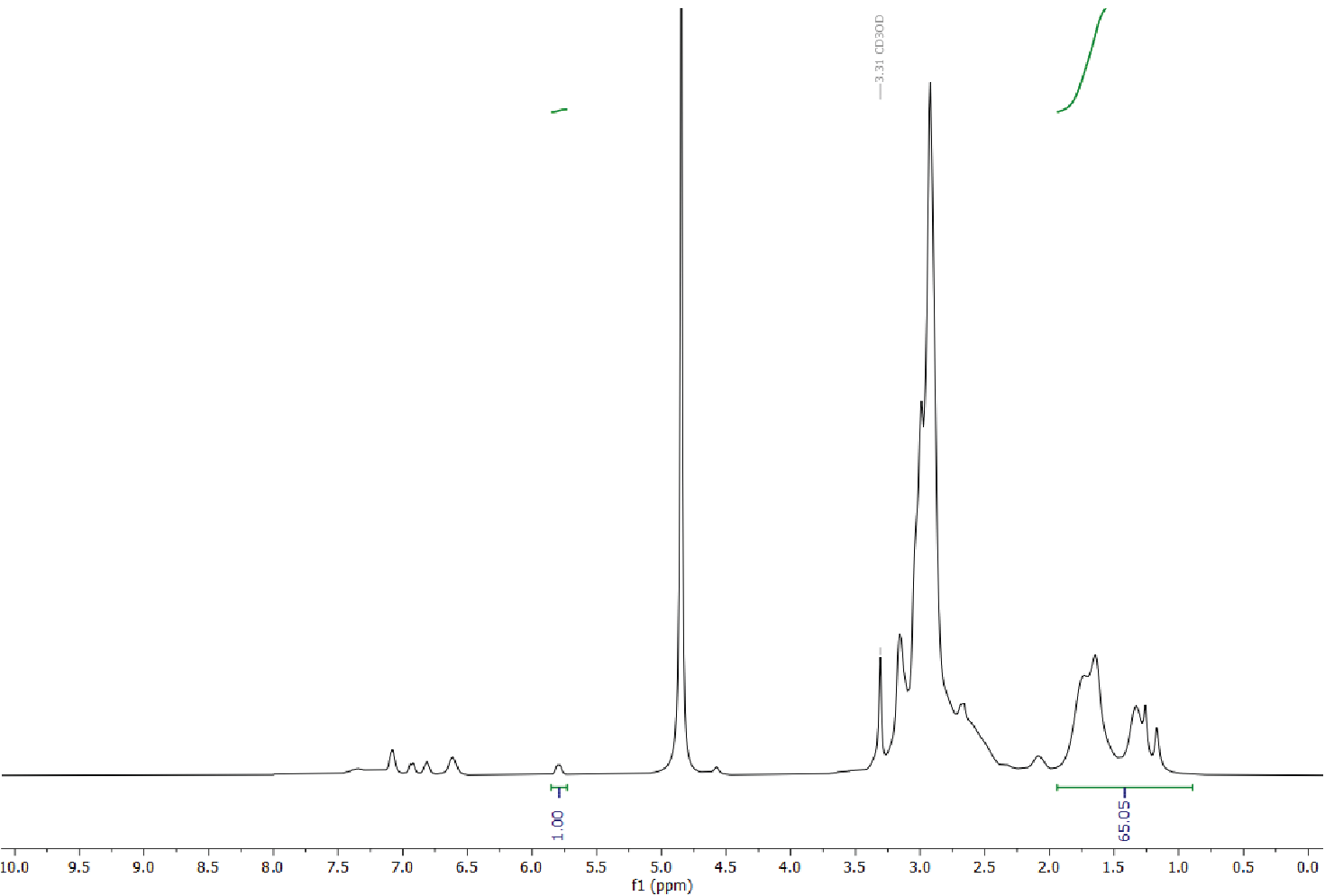


Figure S29: ^1^H-NMR spectra of p-SPLi-2 in CD_3_OD


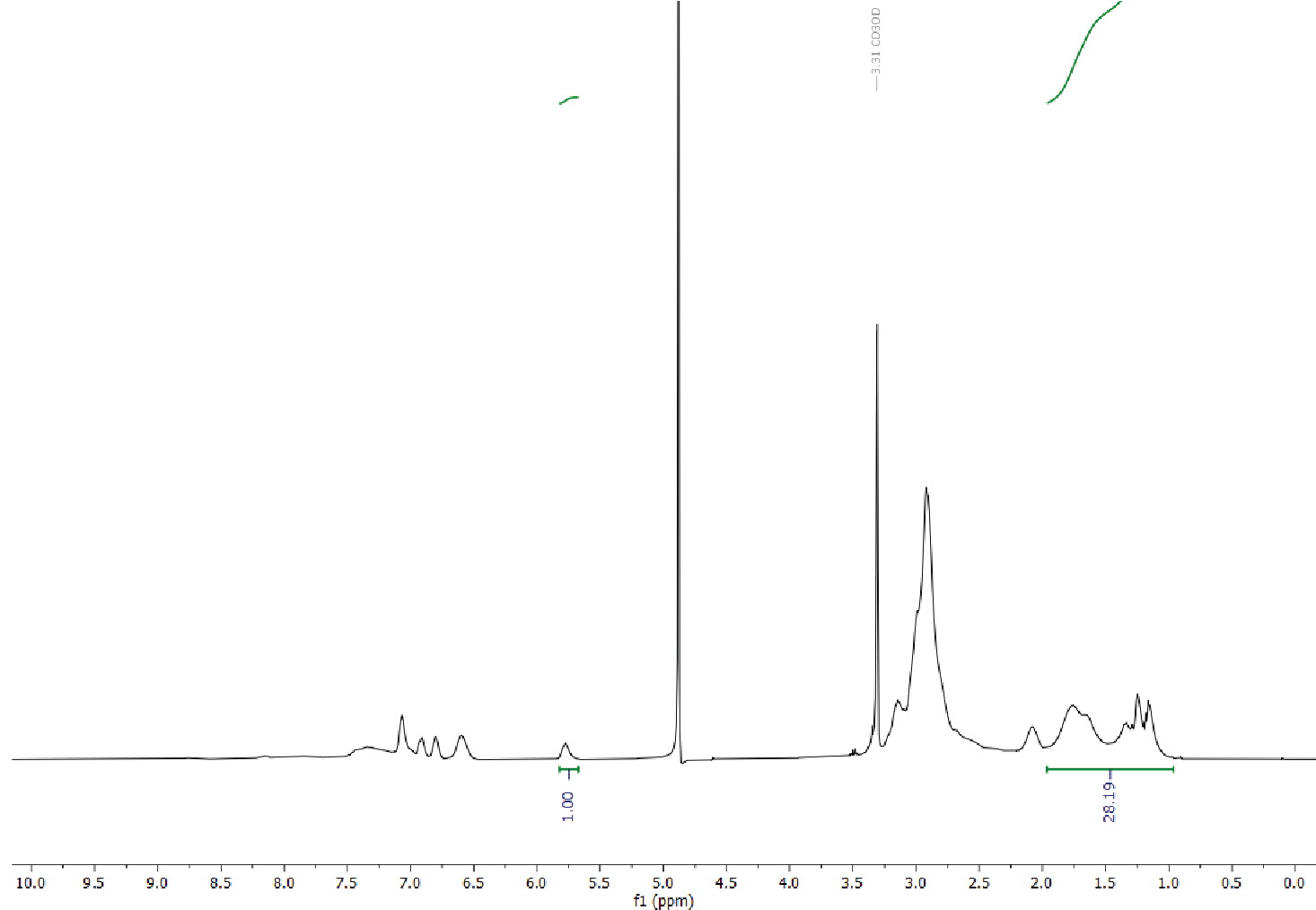

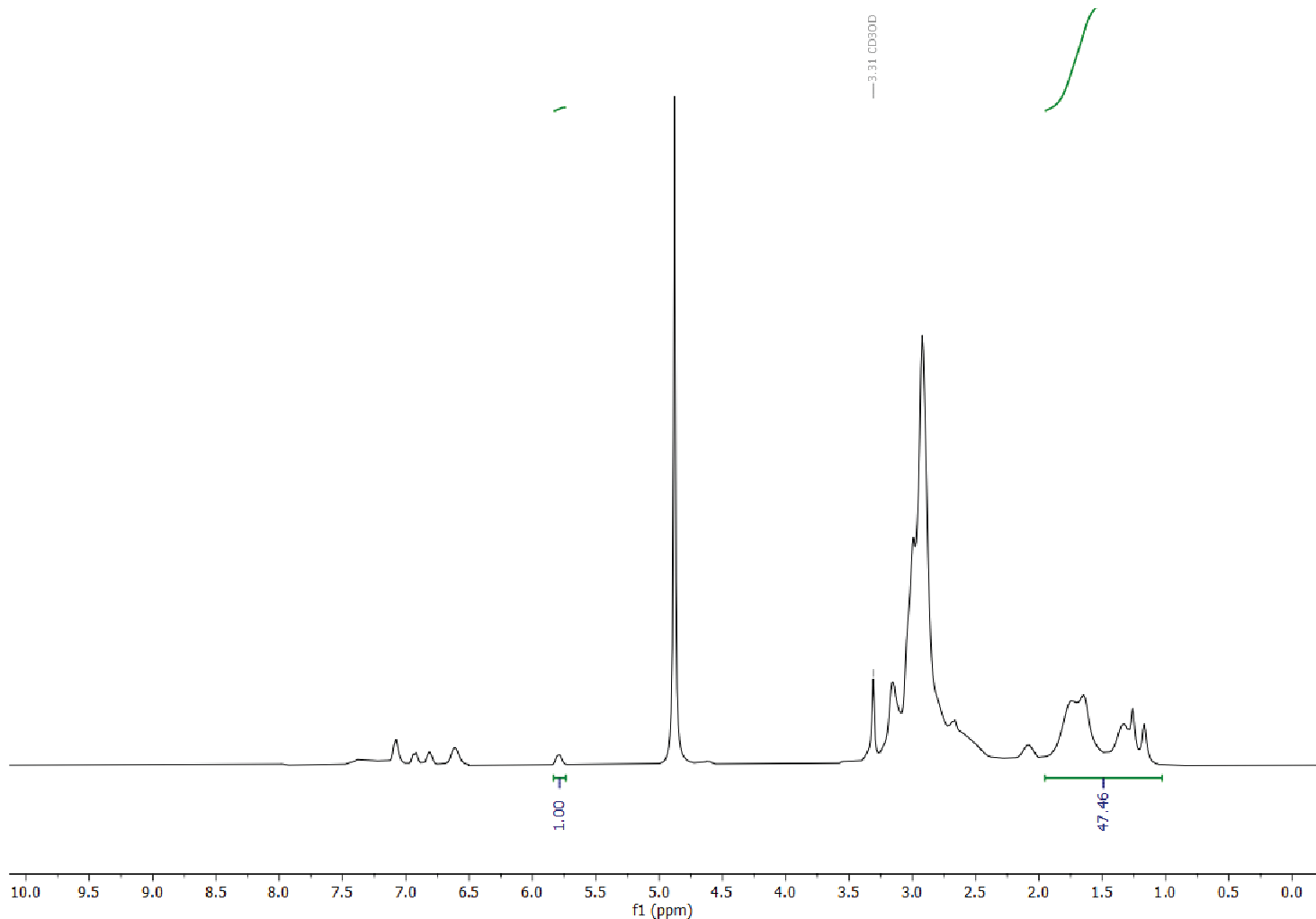
Figure S30: ^1^H-NMR spectra of p-SPLi-3 in CD_3_OD

Figure S31: ^1^H-NMR spectra of p-SPLi-4 in CD_3_OD

10-References

[1] C. Ma, J. Dong, M. Viviani, I. Tulini, N. Pontillo, S. Maity, Y. Zhou, W. H. Roos, K. Liu, A. Herrmann, G. Portale, “De novo rational design of a freestanding, supercharged polypeptide, proton-conducting membrane” *Sci. Adv* **2020**, *6*, 810–827.

[2] E. Epifanovsky, A. T. B. Gilbert, X. Feng, J. Lee, Y. Mao, N. Mardirossian, P. Pokhilko, A. F. White, M. P. Coons, A. L. Dempwolff, Z. Gan, D. Hait, P. R. Horn, L. D. Jacobson, I. Kaliman, J. Kussmann, A. W. Lange, K. U. Lao, D. S. Levine, J. Liu, S. C. McKenzie, A. F. Morrison, K. D. Nanda, F. Plasser, D. R. Rehn, M. L. Vidal, Z. Q. You, Y. Zhu, B. Alam, B. J. Albrecht, A. Aldossary, E. Alguire, J. H. Andersen, V. Athavale, D. Barton, K. Begam, A. Behn, N. Bellonzi, Y. A. Bernard, E. J. Berquist, H. G. A. Burton, A. Carreras, K. Carter-Fenk, R. Chakraborty, A. D. Chien, K. D. Closser, V. Cofer-Shabica, S. Dasgupta, M. De Wergifosse, J. Deng, M. Diedenhofen, H. Do, S. Ehlert, P. T. Fang, S. Fatehi, Q. Feng, T. Friedhoff, J. Gayvert, Q. Ge, G. Gidofalvi, M. Goldey, J. Gomes, C. E. González-Espinoza, S. Gulania, A. O. Gunina, M. W. D. Hanson-Heine, P. H. P. Harbach, A. Hauser, M. F. Herbst, M. Hernández Vera, M. Hodecker, Z. C. Holden, S. Houck, X. Huang, K. Hui, B. C. Huynh, M. Ivanov, Á. Jász, H. Ji, H. Jiang, B. Kaduk, S. Kähler, K. Khistyaev, J. Kim, G. Kis, P. Klunzinger, Z. Koczor-Benda, J. H. Koh, D. Kosenkov, L. Koulias, T. Kowalczyk, C. M. Krauter, K. Kue, A. Kunitsa, T. Kus, I. Ladjánszki, A. Landau, K. V. Lawler, D. Lefrancois, S. Lehtola, R. R. Li, Y. P. Li, J. Liang, M. Liebenthal, H. H. Lin, Y. S. Lin, F. Liu, K. Y. Liu, M. Loipersberger, A. Luenser, A. Manjanath, P. Manohar, E. Mansoor, S. F. Manzer, S. P. Mao, A. V. Marenich, T. Markovich, S. Mason, S. A. Maurer, P. F. McLaughlin, M. F. S. J. Menger, J. M. Mewes, S. A. Mewes, P. Morgante, J. W. Mullinax, K. J. Oosterbaan, G. Paran, A. C. Paul, S. K. Paul, F. Pavošević, Z. Pei, S. Prager, E. I. Proynov, Á. Rák, E. Ramos-Cordoba, B. Rana, A. E. Rask, A. Rettig, R. M. Richard, F. Rob, E. Rossomme, T. Scheele, M. Scheurer, M. Schneider, N. Sergueev, S. M. Sharada, W. Skomorowski, D. W. Small, C. J. Stein, Y. C. Su, E. J. Sundstrom, Z. Tao, J. Thirman, G. J. Tornai, T. Tsuchimochi, N. M. Tubman, S. P. Veccham, O. Vydrov, J. Wenzel, J. Witte, A. Yamada, K. Yao, S. Yeganeh, S. R. Yost, A. Zech, I. Y. Zhang, X. Zhang, Y. Zhang, D. Zuev, A. Aspuru-Guzik, A. T. Bell, N. A. Besley, K. B. Bravaya, B. R. Brooks, D. Casanova, J. Da Chai, S. Coriani, C. J. Cramer, G. Cserey, A. E. Deprince, R. A. Distasio, A. Dreuw, B. D. Dunietz, T. R. Furlani, W. A. Goddard, S. Hammes-Schiffer, T. Head-Gordon, W. J. Hehre, C. P. Hsu, T. C. Jagau, Y. Jung, A. Klamt, J. Kong, D. S. Lambrecht, W. Liang, N. J. Mayhall, C. W. McCurdy, J. B. Neaton, C. Ochsenfeld, J. A. Parkhill, R. Peverati, V. A. Rassolov, Y. Shao, L. V. Slipchenko, T. Stauch, R. P. Steele, J. E. Subotnik, A. J. W. Thom, A. Tkatchenko, D. G. Truhlar, T. Van Voorhis, T. A. Wesolowski, K. B. Whaley, H. L. Woodcock, P. M. Zimmerman, S. Faraji, P. M. W. Gill, M. Head-Gordon, J. M. Herbert, A. I. Krylov, “Software for the frontiers of quantum chemistry: An overview of developments in the Q-Chem 5 package” *Journal of Chemical Physics* **2021**, *155*.
