# Supplementary material for: Photo‐Modulated Proton Transport in Merocyanine Metastable‐State Photoacid Based Polymers
Source: Small. 2026 Feb 25;22(23):e14786. doi: 10.1002/smll.202514786 (PMC13100567; doi:10.1002/smll.202514786)
Supplement: Supplementary file 2 — Supporting File: smll72898‐sup‐0002‐VideoS1.zip. [file SMLL-22-e14786-s001.zip › Legend_Video_1.docx]

**Supplementary Video 1**: Light-induced negative phototaxis of thin hydrogel decorated with monomer 5 (MCH). The bending is caused by the MCH-to-SPH photoisomerization. The light-induced changes in hydrophilicity and in the electrostatic interactions within MCH monomer induces the hydrogel actuation.
